# Genome-Wide Identification and Evolutionary Analysis of the SRO Gene Family in Tomato

**DOI:** 10.3389/fgene.2021.753638

**Published:** 2021-09-21

**Authors:** Ning Li, Ruiqiang Xu, Baike Wang, Juan Wang, Shaoyong Huang, Qinghui Yu, Jie Gao

**Affiliations:** ^1^ College of Forestry and Horticulture, Xinjiang Agricultural University, Urumqi, China; ^2^ Institute of Horticultural Crops, Xinjiang Academy of Agricultural Sciences, Urumqi, China; ^3^ Key Laboratory of Horticulture Crop Genomics and Genetic Improvement in Xinjiang, Urumqi, China

**Keywords:** SRO gene family, tomato, biotic/abiotic stresses, bioinformatics, phylogenetic

## Abstract

SRO (SIMILAR TO RCD ONE) is a family of plant-specific small molecule proteins that play an important role in plant growth and development and environmental responses. However, SROs still lack systematic characterization in tomato. Based on bioinformatics methods, *SRO* family genes were identified and characterized from cultivated tomatoes and several wild tomatoes. qRT-PCR was used to study the expression of *SRO* gene in cultivated tomatoes. Phylogenetic and evolutionary analyses showed that *SRO* genes in angiosperms share a common ancestor and that the number of *SRO* family members changed as plants diverged and evolved. Cultivated tomato had six *SRO* members, five of which still shared some degree of identity with the ancestral *SRO* genes. Genetic structure and physicochemical properties showed that tomato *SRO* genes were highly conserved with chromosomal distribution. They could be divided into three groups based on exon-intron structure, and cultivated tomato contained only two of these subclades. A number of hormonal, light and abiotic stress-responsive *cis*-regulatory elements were identified from the promoter of the tomato *SRO* gene, and they also interacted with a variety of stress-responsive proteins and microRNAs. RNA-seq analysis showed that *SRO* genes were widely expressed in different tissues and developmental stages of tomato, with significant tissue-specific features. Expression analysis also showed that *SRO* genes respond significantly to high temperature and salt stress and mediate the tomato hormone regulatory network. These results provide a theoretical basis for further investigation of the functional expression of tomato *SRO* genes and provide potential genetic resources for tomato resistance breeding.

## Introduction

Plant growth and development are dynamic processes that interact with the surrounding environment. Environmental stress has always been one of the major factors limiting plant growth. The long evolutionary process has endowed plants with many means of coping with biotic and abiotic stresses. Transcription factors, as one of the main ways in which plants regulate their life activities, often play an important role in the plant stress response system (Nevo, 2001; Song et al., 2016). Many key stress response transcription factors have been identified in plants, such as MYB (Du et al., 2009), bHLH (Sun et al., 2018), and WRKY (Li et al., 2020). SRO (SIMILAR TO RCD ONE) is a family of small plant-specific proteins commonly thought to be involved in plant growth and development dynamics and resistance to abiotic stresses (Jaspers et al., 2010a). They are characterized by a C-terminus containing a PARP structural domain involved in a wide range of life activities and an RST structural domain involved in protein-protein interactions, and some SRO members also contain a conserved WWE structural domain associated with the formation of protein globular structures (Jaspers et al., 2010b). *RCD1* was the first member of the SRO family to be discovered and was identified in a yeast 2-hybrid screen using turnip crinkle virus movement protein as bait. *RCD1* is considered to be related to overcoming the oxidative stress-sensitive phenotype of yeast cells (Ahlfors et al., 2004).

Arabidopsis contains 6 SRO family members (*AtSRO1*-*AtSRO6*). *AtSRO1* is a homologous protein with the same domain as *RCD1* and is involved in the plant oxidative stress response and a variety of hormone-induced gene expression systems (Jaspers et al., 2010a). *AtRCD1* loss-of-function mutants are more sensitive to salt stress and osmotic stress and exhibit the characteristics of early flowering and senescence (Overmyer et al., 2000). There is a functional redundancy between *AtSRO1* and *AtRCD1*, whose double mutants have been observed to be severely defective in Arabidopsis embryonic growth and development and have exhibited a pleiotropic phenotype with dwarf plants, short roots and reduced apical dominance (Jaspers et al., 2009; Teotia and Lamb, 2009; Teotia and Lamb, 2011). Overexpression of *AtSRO5* could mediate proline metabolism in Arabidopsis mitochondria, thereby improving plant salt stress and antioxidant capacity (Borsani et al., 2005). *AtSRO2*, *AtSRO3* and *AtSRO5* have shown changes in transcript levels in response to light stress, salt treatment and exposure to O_3_ (Jaspers et al., 2010b; Li et al., 2013), but *AtSRO4* has not yet been reported.

The SRO family has also been characterized in some other species in addition to Arabidopsis. *OsSRO1c* in rice (*Oryza sativa*) is involved in a variety of abiotic stress response processes and interacts with a large number of transcription factors (You et al., 2014). In apple (*Malus domestica*), *MdRCD1* plays a crucial role in the regulation of ROS homeostasis. Its ectopic expression significantly enhances the resistance of transgenic lines to salt and oxidative stress (Li et al., 2017). All *ZmSROs* in maize (*Zea mays*) are specifically expressed in the roots and respond to high salt and drought stress to varying degrees (Jiang et al., 2018). The 30 *TaSRO* members in wheat (*Triticum aestivum*) are divided into two different groups. Most *TaSROs* are highly expressed in one or more tissues, participate in the wheat hormone regulation network and are induced by the wheat stress response (Jiang et al., 2020). Banana (*Musa nana*) contains 6 *MaSROs*, which actively respond to biotic/abiotic stresses by mediating a hormone regulatory network. *MaSRO4* could interact with *MaNAC6* and *MaMYB4* through the PARP domain to regulate downstream signalling pathways (Zhang et al., 2019). The above studies have shown that the SRO family participates in a variety of plant stress responses and regulates the processes of plant growth and development.

Tomato is the largest vegetable cash crop widely planted in the world and is favoured by consumers worldwide. However, tomato cultivation still has not eliminated the effects of biotic and abiotic stress. Every year, billions of tomato yield are lost due to adverse stress (Krishna et al., 2019). Tomato is rich in genetic diversity. Wild tomato usually has strong stress resistance and extremely rich variation. It has advantages over cultivated tomatoes in resisting biotic and abiotic stresses (Lin et al., 2014; Szymański et al., 2020). Studying the response dynamics of wild tomato to adverse environments can provide an important theoretical basis and genetic resources for research on the stress tolerance of cultivated tomato. Although there is evidence that the *JWS-26* gene, which is similar to the *AtSRO5* sequence, is significantly upregulated in tomato roots under salt stress (Babajani et al., 2009), systematic studies on the *SRO* gene family of tomato have not yet been reported. In this study, we used bioinformatics methods to comprehensively identify the *SRO* gene families in cultivated tomato (*S. lycopersicum, S. lycopersicum* var. *cerasiforme*) and wild relatives (*S. pennellii*, *S. pimpinellifolium*, *S. chilense*, and *S. lycopersicoides*). The physical and chemical properties, gene structure, evolutionary characteristics and functional expression of the SRO family were analysed, and the unregulated mechanism of the SRO family in tomato in response to different stresses was discussed. This study provides a basis for clarifying the function of the SRO protein and provides a theoretical reference for stress gene mining and breeding of cultivated tomato.

## Materials and Methods

### Plant Materials and Growth Conditions

The plant materials used in this study were tomato cultivars (*Solanum lycopersicum*, M82) from our laboratory. Tomatoes were grown in a 24 ± 2°C common greenhouse under a 16 h light/8 h dark photoperiod, and the relative humidity was 60–70%. Four-week-old seedlings were used for stress and hormone treatments. Salt stress was applied to seedlings treated with 150 mM sodium chloride (NaCl), and seedlings were transferred to a growth chamber at 40°C to simulate heat shock stress. Leaves were collected after 0, 2, 4 and 8 h for the stress treatments. Seedlings were sprayed with 100 µM IAA, 100 µM MeJA or 100 μM ABA, and tomato leaves were collected after 0, 6, 12 and 24 h. The isolated tissues were frozen in liquid nitrogen and then transferred to −80°C. Three different biological sample sources were collected for subsequent experiments in each process.

### Identification of *SRO* Genes in Multiple Species

Complete genome sequences of grape and coffee were downloaded from the Ensemble Plants database (https://plants.ensembl.org/index.html). The reported amino acid sequences of Arabidopsis atrcd1 and *AtSRO*1-5 (Jaspers et al., 2010a) were downloaded from The Arabidopsis Information Resource (TAIR: https://www.arabidopsis.org/) (Rhee et al., 2003). The genomes of the major Solanaceae plants were downloaded from the Solanaceae genome database (https://solgenomics.net/), and *AtSROs* were used as query sequences for the whole genome sequence BLASTP search in the Phytozome database (https://phytozome.jgi.doe.gov) to extract *SRO* members from various plants (Goodstein et al., 2012; Fernandez-Pozo et al., 2015). Similarly, BLASTP was used to search the local *Solanaceae* plant protein database (E-value: 1e^−5^) for *AtSROs* PFAM database (http://pfam.xfam.org/) was used to download Hidden Markov Models for RST (PF12174), PARP (PF00644) and WWE (PF02825) domains (Bateman et al., 2004; Sonnhammer et al., 1997). The canonical domains were used to Hmmsearch (Finn et al., 2011) from the local *Solanaceae* protein database with HMMER 3.0 (E-value: 1e-5). All candidate gene domains were analysed in smart (http://smart.embl.de/), CDD search (HTTPS://www.ncbi.NLM.NIH.Gov/CDD/) and Pfam (http://pfam.xfam.org/) databases (Sonnhammer et al., 1997; Schultz et al., 2000; Marchler-Bauer et al., 2007). The *SRO* genes in *Solanaceae* were obtained by deleting the genes without any typical SRO family domains and retaining a representative transcript of each gene (Supplementary Data S1).

The ExPASy online database ProtParam tool (http://www.expasy.org/protparam/) was used to predict and analyse the amino acid number (Artimo et al., 2012), isoelectric point, fat index and other physical and chemical properties of the tomato SRO protein. Protein subcellular localization was predicted by WoLF PSORT Online software (https://wolfpsort.hgc.jp/) (Horton et al., 2007).

### Construction of Conserved Motifs, *Cis*-regulatory Elements and Phylogenetic Tree of *SRO* Genes in Tomato

Meme software (v4.12.0) was used to search tomato *SRO* motifs (Grundy et al., 1997); the number of searches was 20, the maximum and minimum widths were set to 6 and 50, respectively. Tbtools was used to draw conservative motifs and gene structure maps (Chen et al., 2020). According to the position information of the *SRO* gene on the chromosome, the karyotype map of tomato was drawn using mapchart. MEGA 7.0 software was used for multiple sequence alignment, and the maximum likelihood (ML) and neighbour joining methods were used to construct the phylogenetic tree with Poisson correction (Kumar et al., 2016). The bootstrap value was set to 2000. The Itools online website (https://itol.embl.de/) was used to display the midpoint rooted base tree. The promoter sequence of the *SRO*genes in tomato (2000 bp upstream of the translation start point) was extracted, and the *cis*-regulatory element (CRE) of the *SRO* genes was predicted through the Search for CARE tool in the PlantCARE database (http://bioinformatics.psb.ugent.be/webtools/plantcare/html/) (Rombauts et al., 1999) GSDS (http://gsds.gao-lab.org/) Online software was used to draw a distribution map of CREs (Hu et al., 2015).

### Tomato *SRO* Family Homologous Genes, Interaction Network Andexpression Analysis

Perl scripts were used to extract the *SRO* gene position on the chromosome, and McscanX was used to extract the collinearity relationship between *SRO* genes (Wang et al., 2012). The substitution rate of paralogous genes was calculated by KaKs_Calculator2.0 (Wang et al., 2010), and Tbtools was used to draw the collinearity analysis map of orthologous genes of each species. The protein-protein interaction relationship was predicted by the STRING online website (https://string-db.org/), and the microRNA targeting relationship was predicted by psRNATarget (http://plantgrn.noble.org/psRNATarget/) with default parameters (Dai et al., 2018; Szklarczyk et al., 2019). The interaction network was displayed by Cytoscape software (Su et al., 2014).

The expression data of *SRO* genes in different tissues and developmental stages, inculding leaves, roots, flower buds, fully opened flowers, 1 cm fruits, 2 cm fruits, 3 cm fruits, mature green fruits, breaker fruits and breaker + 10 days fruits, were retrieved from the Tomato Functional Genomics database (TFGD, http://ted.bti.cornell.edu/) (Fei et al., 2011). Seedings of M82 (salt-sensitive) and *S. pennellii* (elite salt-resistant) were exposed to salt stress (200 mM NaCl, Irrigation) after 6 weeks of normal growth, 0 and 12 h tomato roots were used for RNA-seq in illumina Hiseq 2500 platform. The expression level were normalized by Transcripts Per Million (TPM). The R package DESeq2 (Love et al., 2014) was then used to calculate the Fold Change (FC). All *SRO* genes expression profiles were analyzed and performed using software Tbtools. The raw data were deposited in the Genome Sequence Archive (GSA) of the China National Center for Bioinformation under accession number: PRJCA005251 (unpublished).

### Ribo Nucleic Acid Extraction and Reverse Transcription Polymerase Chain Reaction Analysis

Total RNA was extracted using TRIzol reagent (Aidlab Biotechnologies, Beijing, China). First-strand cDNA was synthesized using a HiScript II 1st Strand cDNA Synthesis Kit (+gDNA wiper) (Vazyme, China). Gene-specific primers were designed using Primer Premier 5.0 (Supplementary Table S1), and the primers for these genes were synthesized by Sangon Biotech Co., Ltd. (Shanghai, China). Then, quantitative PCR (qPCR) was performed using Maxima SYBR Green/ROX qPCR Master Mix. The EF-1α gene was used as an internal reference. Each treatment contained three independent biological replicates, and each replicate contained three technical replicates. Gene expression was calculated using the 2^−ΔΔCt^ method (Livak and Schmittgen, 2001).

## Result

### Identification of the *SRO* Genes in Tomato

In this study, we used *Arabidopsis thaliana* amino acid sequences (*AtSROs*) for BLASTP and HMM searches (RST, PARP and WWE) to screen *SRO* members with at least one conserved domain in the genomes of multiple tomatoes and named them according to their positions on chromosomes. The cultivated tomatoes contained 6 *SRO* genes. The number of *SRO* family genes in the wild tomatoes was 7–11. Analysis of protein physicochemical properties showed that the length of the *SRO* family amino acids in all tomatoes ranged from 217 (*SpenSRO5*) to 680 (*SlycSRO4*), the molecular weight ranged from 24569.40 (*SpenSRO5*) to 77592.90 (*SlycSRO4*), the pI ranged from 5.57 (*SlydSRO2*) to 9.58 (*SpenSRO5*), the aliphatic index of the SRO protein ranged from 62.93 (*SolySRO3*) to 92.53 (*SpenSRO5*), and the GRAVY value ranged from −0.12 to −0.48 (Table 1). Chromosome localization (Supplementary Figure S1) showed that the *SRO* family in tomato is distributed in 7 regions on 6 chromosomes. The *SRO* genes on Chr1 and Chr4 in cultivated tomato were lost. Subcellular localization showed that the *SRO* genes on Chr1, Chr4, and Chr5 were distributed in the cytoplasm and chloroplast, and the rest of the *SRO* was located in the nucleus (Table 1).

**TABLE 1:** Basic information of *SRO* genes identified in tomato.

Species	Gene id	Gene name	Chr	Length	MW (Da)	pI	Aliphatic index	GRAVY	Subcellular localization
*S. lycopersicum*	Solyc03g114360	*SolySRO1*	Soly-3	375	41317.55	8.61	69.15	−0.40	nucl
	Solyc05g005280	*SolySRO2*	Soly-5	304	34189.18	7.65	84.31	−0.28	cyto
	Solyc05g005290	*SolySRO3*	Soly-5	233	26286.90	6.71	62.93	−0.25	cyto
	Solyc06g066330	*SolySRO4*	Soly-6	595	67309.50	7.67	85.61	−0.43	nucl
	Solyc08g005270	*SolySRO5*	Soly-8	600	67858.03	6.39	73.70	−0.45	nucl
	Solyc08g076420	*SolySRO6*	Soly-8	598	67307.56	7.20	81.09	−0.44	nucl
*S. lycopersicum* var. *cerasiforme*	SLYcer01g04782	*SlycSRO1*	Slyc-1	300	34191.15	7.70	83.43	−0.39	nucl
	SLYcer01g04783	*SlycSRO2*	Slyc-1	442	50504.70	6.44	82.42	−0.37	cyto
	SLYcer03g04627	*SlycSRO3*	Slyc-3	376	41417.66	8.60	68.96	−0.41	nucl
	SLYcer04g05316	*SlycSRO4*	Slyc-4	680	77592.90	5.82	85.94	−0.36	nucl
	SLYcer04g05317	*SlycSRO5*	Slyc-4	483	55418.48	6.36	84.72	−0.43	cyto
	SLYcer04g05318	*SlycSRO6*	Slyc-4	443	49038.35	6.54	84.83	−0.18	chlo
	SLYcer05g00116	*SlycSRO7*	Slyc-5	315	35392.43	6.60	83.52	−0.31	cyto
	SLYcer05g00117	*SlycSRO8*	Slyc-5	320	36137.59	8.91	85.59	−0.29	cyto
	SLYcer06g04350	*SlycSRO9*	Slyc-6	594	67352.60	7.67	85.27	−0.44	nucl
	SLYcer08g00172	*SlycSRO10*	Slyc-8	600	67888.60	6.81	73.70	−0.45	nucl
	SLYcer08g05857	*SlycSRO11*	Slyc-8	507	57279.60	6.70	80.89	−0.48	nucl
*S. chilense*	SOLCI001453300	*SolcSRO1*	—	233	26254.13	7.08	89.14	−0.23	cyto
	SOLCI001453400	*SolcSRO2*	—	315	35419.46	6.60	83.84	−0.32	cyto
	SOLCI001464200	*SolcSRO3*	—	594	67595.43	7.54	86.50	−0.44	nucl
	SOLCI003930500	*SolcSRO4*	—	600	67793.00	6.90	76.63	−0.41	nucl
	SOLCI004134700	*SolcSRO5*	—	597	67250.52	6.69	81.41	−0.43	nucl
	SOLCI005404200	*SolcSRO6*	—	310	34869.80	6.21	85.45	−0.30	nucl
	SOLCI005589700	*SolcSRO7*	—	375	41302.52	8.63	69.50	−0.41	nucl
S. *pimpinellifolium*	SPI01g04931	*SpiSRO1*	Spi-1	442	50525.01	6.33	84.19	−0.37	cyto
	SPI03g04680	*SpiSRO2*	Spi-3	376	41417.00	8.70	68.96	−0.41	nucl
	SPI04g04808	*SpiSRO3*	Spi-4	679	77360.60	5.82	86.49	−0.34	nucl
	SPI04g04809	*SpiSRO4*	Spi-4	483	55194.90	6.83	83.91	−0.41	cyto
	SPI04g04810	*SpiSRO5*	Spi-4	443	49197.44	6.20	85.28	−0.20	chlo
	SPI05g00126	*SpiSRO6*	Spi-5	315	35392.39	6.41	83.84	−0.31	cyto
	SPI05g00127	*SpiSRO7*	Spi-5	320	36246.71	8.81	85.59	−0.31	cyto
	SPI06g04210	*SpiSRO8*	Spi-6	594	67352.60	7.67	85.70	−0.44	nucl
	SPI08g00091	*SpiSRO9*	Spi-8	600	67856.50	6.81	74.80	−0.44	nucl
	SPI08g05744	*SpiSRO10*	Spi-8	507	57285.60	6.51	81.60	−0.47	nucl
S. *pennellii*	Sopen03g033460	*SpenSRO1*	Spen-3	375	41250.55	8.73	70.96	−0.39	nucl
	Sopen04g030720	*SpenSRO2*	Spen-4	595	66837.97	5.74	81.98	−0.39	nucl
	Sopen04g030730	*SpenSRO3*	Spen-4	455	50100.49	5.94	88.79	−0.12	chlo
	Sopen05g001280	*SpenSRO4*	Spen-5	315	35408.45	6.51	83.84	−0.28	cyto
	Sopen05g001300	*SpenSRO5*	Spen-5	217	24569.40	9.58	92.53	−0.19	cyto
	Sopen06g021690	*SpenSRO6*	Spen-6	594	67113.79	7.56	85.44	−0.42	nucl
	Sopen08g001290	*SpenSRO7*	Spen-8	595	67457.00	7.09	75.60	−0.44	nucl
	Sopen08g025000	*SpenSRO8*	Spen-8	597	67299.00	7.62	80.75	−0.44	nucl
S. *lycopersicoides*	Solyd03g075660	*SlydSRO1*	Slyd-3	352	38871.74	8.79	67.87	−0.42	nucl
	Solyd05g050320	*SlydSRO2*	Slyd-5	376	41703.19	5.57	75.48	−0.30	nucl
	Solyd06g065810	*SlydSRO3*	Slyd-6	594	67162.05	8.50	85.62	−0.40	nucl
	Solyd08g050330	*SlydSRO4*	Slyd-8	539	60715.62	5.72	73.75	−0.42	nucl
	Solyd08g050340	*SlydSRO5*	Slyd-8	600	67979.41	6.50	74.70	−0.42	nucl
	Solyd08g068000	*SlydSRO6*	Slyd-8	597	67197.55	6.50	82.06	−0.39	nucl

### Phylogenetic Analysis of *SRO* Genes in Various Plants

**FIGURE 1:**
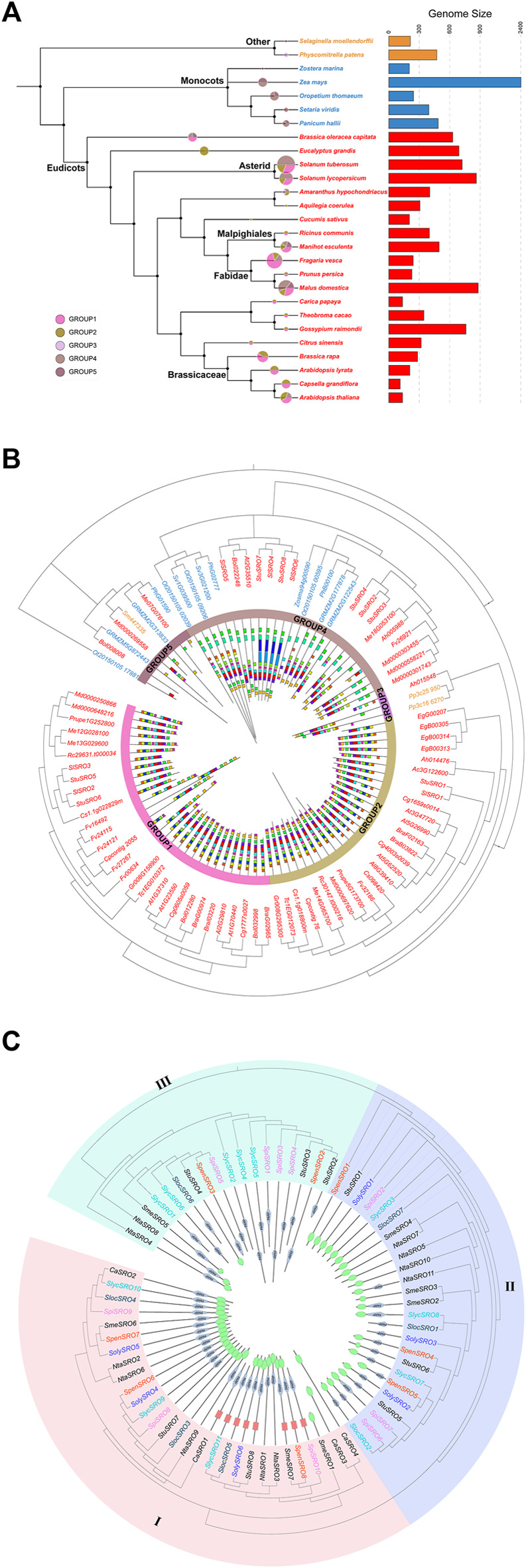
Comparison of the SRO family in plants. **(A)** Evolutionary relationships and number of SRO families in multiple species, with species colours representing their Taxonomic characteristics and the size and colour of the sectors representing the number of SRO family in the species and the subgroups to which they belong. **(B)** Phylogenetic trees were constructed for 93 *SRO* genes using the NJ method. Different colors represent species with different taxonomic characteristics. Gene structure and conserved motif were peformed inside the phylogenetic tree. **(C)** Phylogenetic tree of the *SRO* family in Solanaceae. The phylogenetic tree was constructed using the NJ method. The different coloured *SRO* genes were derived from different tomato species, and the conserved structural domains of the corresponding *SRO* genes are shown inside the evolutionary tree, with the WWE structural domain in red, the RST structural domain in green, and the **PARP** structural domain in grey.

The Arabidopsis Information Resource (TAIR), PlantGDB, Phytozome, and National Center for Biotechnology Information (NCBI) databases were used to retrieve reliable *SRO* sequences (Li et al., 2019), and 93 *SRO* potential homologous genes were retrieved from 27 plants (20 Eudicots, 5 Monocots, 1 Bryophyta, and 1 Tracheophyta). The *SRO* gene family of plants evolved continuously with the evolution of the complexity of life (Figure 1A). There were obvious taxonomic differences among Bryophytes, Tracheophytes, Monocots and Eudicots, but the expansion of the *SRO* family was relatively conservative, although the number of *SRO* genes in Asterid, Fabidae and Brassicaceae was significantly higher than that in *Physcomitrella patens* and *Selaginella moellendorffii*. However, the *SRO* families in some higher plants seemed to be under more selection pressure, and the number of genes was reduced. The amino acid sequences of 93 *SRO* homologous genes were used to construct the evolutionary tree by the neighbour joining method (Figure 1B). All SRO families were divided into 5 groups, among which GROUP1 and GROUP2 contained only Eudicots, GROUP3 contained only *P. patens* and *A. hypochondriacu* ***s***, and GROUP4 and GROUP5 contained both Eudicot and Monocot plants. Conserved motif analysis showed that each group exhibited higher similarities. Eudicots accumulated more subfamily types than Monocots. These results indicated that the expansion of SRO family members coincided with whole-genome duplication (WGD) during plant evolution.

To further analyse the lineage-specific amplification of the *SRO* family, we identified the *SRO* family in *Solanaceae* (*Capsicum annuum*, *Solanum melongena*, *Solanum tuberosum*, *Nicotiana tabacum*, *Solanum lycopersicum*, and *Lycopersicon*) and constructed a phylogenetic tree (Figure 1C). All *SRO* genes were divided into three classes. There were large differences in coding sequence (CDS) length and domain among them. The genes in class I showed the longest gene length and contained both PARP and RST domains, with the exception of *SmeSRO1*, *CaSRO*3 and *CaSRO*4. Some class I genes also contained the WWE domain. The length of genes in class II was the shortest, and some of them only contained the RST domain. Among the class III genes, *NitaSRO4*, *NitaSRO8* and *SmeSRO5* contained a small RST domain at the N-terminus, and the other genes only contained the PARP domain. *S. lycopersicum* in particular was lost in class III. In fact, these *SRO* genes were mainly located on Chr1 and Chr4 of their respective species, which was consistent with the results of the chromosome localization map. We noticed that the four *SRO* genes (*CaSRO1 ∼ 4*) identified in *C. annuum* were all classified in region I of the phylogenetic tree and were lost in particular in class II and class III. The domains of *CaSRO*3 and *CaSRO*4 were different from the others in class I.

### Structure and Conserved Motif Analysis of *SRO* Genes in Tomato

**FIGURE 2:**
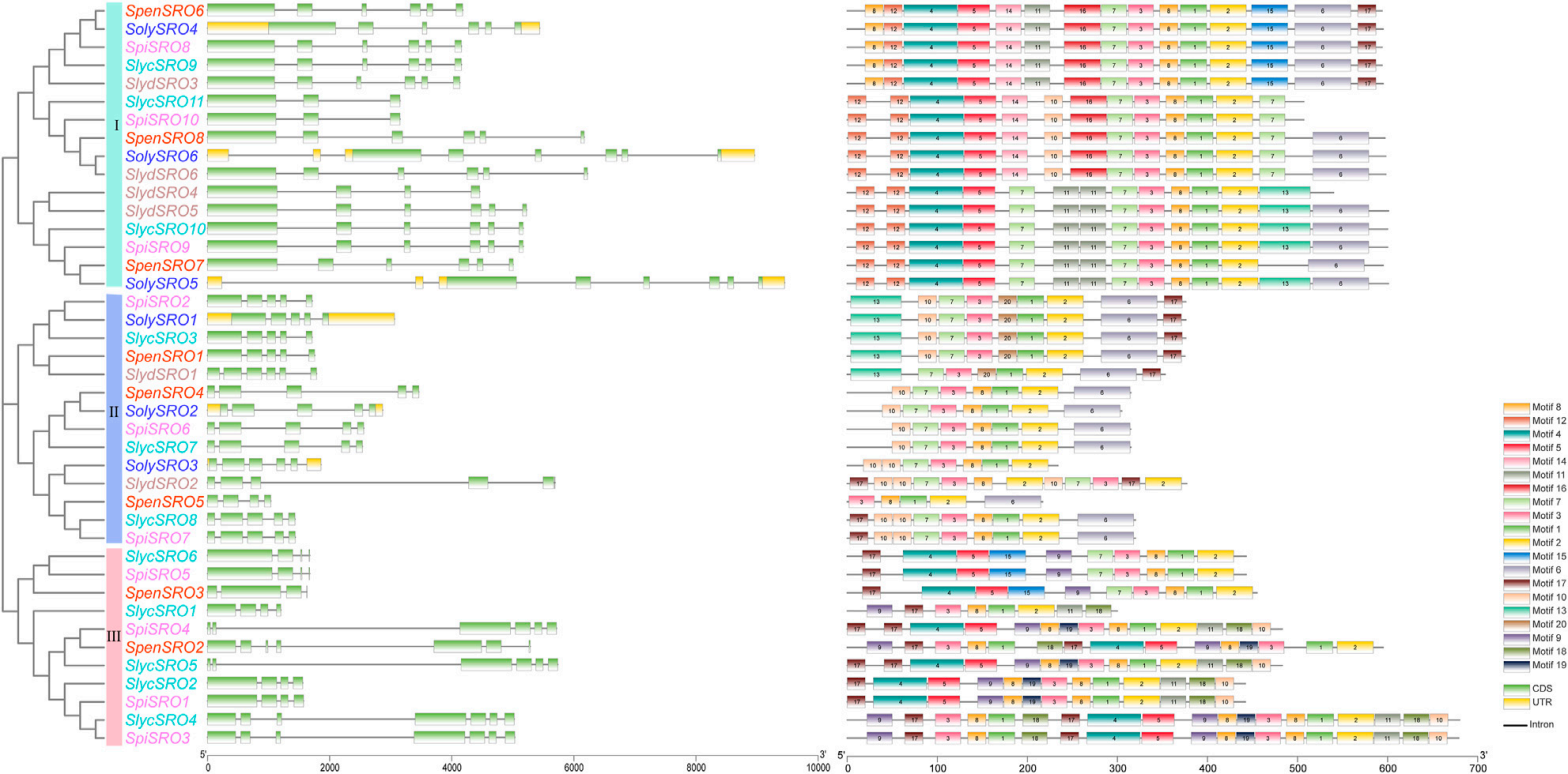
Phylogenetic relationships, Structure and conserved motifs of *SRO* genes in tomato species. The different coloured *SRO* genes were derived from different tomato species. Green boxes indicate exons, yellow boxes indicate UTR and black lines indicate introns. The numbers 1–20 and the different colored boxes indicate motifs.

Exon-intron structural differences are important sources of gene family variation and plant biodiversity. Different structures determine the differential function and expression of genes (Xu et al., 2012). Except for *S. chilense,* whose *SRO* genes were not assembled on the chromosome, we extracted all *SRO* gene annotations from the whole genomes of cultivated tomato and multiple wild tomatoes. The comparison results of the positions and quantity of exons were visualized with TBtools (Figure 2). The results of the phylogenetic tree showed that all *SRO* genes were divided into three groups, among which, in group I, *SlycSRO11* and *SpiSRO10* contained three exons, *SlydSRO4* contained 4 exons, and the rest of the *SRO* genes contained 6 exons. In group II, *SlydSRO1* contained 6 exons, *SpenSRO5* contained 4 exons, and all other *SRO* genes contained 5 exons. Obviously, the length and structure of the *SRO* genes in groups I and II were relatively consistent. We noticed that even with the same number of exons, *SRO* genes of cultivated tomato in groups I and II still exhibited more introns and longer gene lengths than those of wild tomatoes. Group III, which contained only wild tomato, showed more structural diversity. The first *SRO* genes (*SpenSRO2*, *SlycSRO4*, and *SpiSRO3*) on Chr4 of all wild tomatoes showed higher structural similarity; they had 7 exons and almost the same gene length. The *SRO* genes (*SlycSRO1* and *SpiSRO1*) on Chr1 and the third *SRO* genes (*SlycSRO2*, *SlycSRO6*, *SpiSRO5*, and *SpenSRO3*) on Chr4 showed similar regularity. They had the same length and four exons. Only *S. lycopersicum* var. *cerasiforme* and *S. pimpinellifolium* contained the second *SRO* genes (S*lycSRO5* and *SpiSRO4*) on Chr4, and they had seven exons with the same distribution.

The conserved motifs of all *SRO* genes were predicted based on MEME software, and a total of 20 conserved motifs were identified (Supplementary Figure S2). Motif6 and motif8 are the RST and PARP domains, respectively, and they were distributed in all *SRO* genes. Similar to the exon-intron structure, the conserved motifs were also divided into three groups based on genetic relationships. The motif composition of the *SRO* gene in the same groups was similar. Group I contained the largest number of motifs, with a total of 16 motifs. Motif12, motif14 and motif16 only appeared in this group. Group II contained 11 motifs, including motif20, and group III contained 14 motifs, including motif9, motif18 and motif19. The *SRO* genes of cultivated tomato also only appeared in groups I and II, and each *SolySROs* was always genetically close to one or more *SROs* in wild tomatoes. The *SRO* gene motifs on the same branch cluster were highly similar in both cultivated and wild tomatoes, indicating that there were no significant differences in the sequence and function of *SRO* genes in tomato species, with the exception of group III.

### Promoter Analysis of *SRO* Genes in Tomato

**FIGURE 3:**
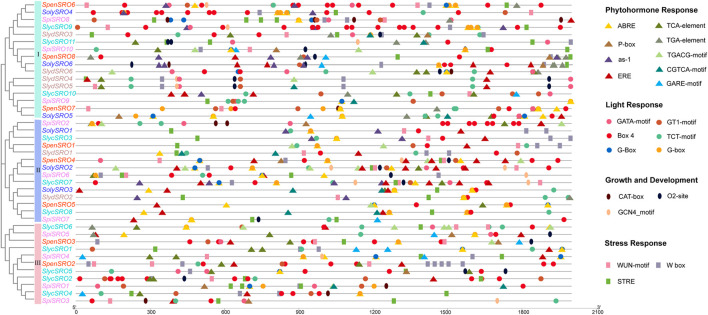
Distribution of CREs of *SRO* genes in tomato. The different coloured *SRO* genes were derived from different tomato species. Different CREs were indicated by different shapes, triangles indicate hormone response elements, circles indicate light response elements, ellipses indicate growth and development related elements, boxes indicate stress response related elements, and different elements were indicateed by different colors.

CRE control gene expression by combining with specific transcription factors, and the distribution of CREs in the promoter region is closely related to gene function (Biłas et al., 2016). We predicted the CRE in the 2000 bp sequence upstream of all *SRO* genes through the Plantcare online website (Figure 3, Supplementary Table S2). In addition to the core promoter and enhancer, the promoter region of *SRO* genes in tomato contained a large number of plant hormone response elements. A total of 543 plant hormone response elements were identified and divided into 20 species, including 190 abscisic acid response elements of 5 types, 112 salicylic acid response elements of 4 types, 34 gibberellin response elements of 3 types, 33 auxin response elements of 3 types, 118 methyl jasmonate response elements of 2 types, 56 ethylene response elements of one type. Two auxin response elements (AuxRR-core, E2Fb) and one salicylic acid response element (SARE) were only specifically recognized in wild tomato. There were the mosttypes of light-responsive elements. Among all *SRO* genes, 23 types of light-responsive elements were identified, a total of 452, mainly including 102 conserved DNA modules involved in the Box4 light response, 73 light-induced stem- and leaf-specific expression promoter G-boxes, 48 photosynthetic element TCT motifs induced by sunlight time, and 45 photosyntheticelement GT1 motifs. Of these elements, 7 types of light-responsive elements (AAAC motif, AT1 motif, ATCT motif, chs-CMA2a, gap box, LAMP element and Sp1) were only specifically identified in wild tomatoes. Nine types of biotic/abiotic stress response elements were identified, for a total of 285, including 84 anaerobic inducing elements (AREs), 45 drought response elements (W-boxes), 48 high temperature response elements (STREs), and 37 wound inducing elements (WUN motifs). Sixty-seven growth and development response elements were also identified in all *SRO* genes, divided into 8 types, including 12 CAT boxes related to meristem expression, 13 GCN4 motifs related to endosperm expression, and 23 O2 sites participating in zein metabolism regulation. Among them, four growth and development response elements (AACA motif, CCGTCC box, HD-Zip 1, and MSA-like) were lost in cultivated tomato. At the same time, *SRO* genes in tomatoes also contained a large number of other regulatory elements. These results indicated that *SRO* genes were widely involved in various life activities, such as plant growth and development and stress responses.

### Duplication Gene and Ka/Ks Analysis of *SRO* Genes in Tomato

Gene replication is an effective way for organisms to obtain new genes and maintain gene vitality (Zhang, 2003). Local blast and mcscanx software were used to extract the repeat sequences of the *SRO* gene in all tomato genomes, and the replacement rate of *SRO* homologous gene pairs was calculated using KaKs Calculator 2.0 (Table 2). The results showed two paralogous gene pairs in the *SRO* family of cultivated tomato, namely, *SolySRO2*/*SolySRO3* and *SolySRO4*/*SolySRO6*, and all were derived from segmental replication. *S. pennellii*, *S. Chilense* and *S. lycopersicoides* also contained two pairs of paralogous genes, while *S. pimpinellifolium* and *S. lycopersicum* var. *cerasiforme* contained 6 and 11 pairs of *SRO* paralogous gene pairs, respectively, which mainly came from multiple repeat pairs of the *SRO* gene on Chr1 (Slyc1, Slyc2, and Spi1) and Chr 4 (Slyc4, Slyc5, Slyc6, Spi3, and Spi4). In all wild tomatoes, *SlycSRO1*/*SlycSRO2*, *SlycSRO7*/*SlycSRO8*, *SpiSRO3*/*SpiSRO4* and *SpiSRO6*/*SpiSRO7* paralogue gene pairs were derived from chromosome tandem replication, and the rest of the repeat gene pairs were derived from segmental replication. The *Ka*/*Ks* of the two homologous gene pairs in cultivated tomato were both <1, indicating that the two pairs of paralogous genes had received strong environmental pressure, and the gene evolution and protein function had stabilized. There were still 9 pairs of paralogous genes *Ka*/*Ks* greater than 1 in wild tomatoes. These *SRO* genes were subjected to positive environmental selection and were still in the rapid evolutionary stage. According to the differentiation rate R (1.5 × 10^−8^) of Solanaceae (Blanc and Wolfe, 2004), the differentiation time of all gene pairs was estimated. The duplication time of the *SRO* paralogous gene pairs in tomato was more dispersed, ranging from 5.62 to 45.33 Mya. Duplication of the *SolySRO2*/*SolySRO3* fragment on cultivated tomato chromosome 5 occurred at approximately 12.99 Mya. However, the homologous gene pairs of *SpenSRO4*/*SpenSRO5* and *SpiSRO6*/*SpiSRO7*, which were also distributed on chr5, replicated at 45.33 and 5.62 Mya in segmental and tandem manners, respectively. Duplication of the *SolySRO4*/*SolySRO6* homologous gene pair occurred at approximately 38.68 Mya, and duplication of homologous genes in the same region in *S. pimpinellifolium* and *S. lycopersicoides* occurred at 35.30 and 36.21 Mya, respectively. These three homologous gene pairs were relatively close in duplication time, while their *Ka*/*Ks* values converged to 1, which could mean that the *SRO* genes of tomatoes on Chr6 and Chr8 occurred early after whole genome duplication in Solanaceae, and these genes belong to the conserved members of the *SRO* family.

**TABLE 2:** The *Ka*/*Ks* ratios and date of duplication for duplicate *SRO* genes in tomato.

Species	Chr	Duplicated gene pairs	Ka	Ks	Ka/Ks	Selective pressure	Type	Time (Mya^a^)
*S. lycopersicum*	Soly5/Soly5	*SolySRO2*/*SolySRO3*	0.13	0.39	0.32	Purify selection	segmental	12.99
	Soly6/Soly8	*SolySRO4*/*SolySRO6*	0.97	1.16	0.83	Purify selection	segmental	38.68
*S. pennellii*	Spen4/Spen4	*SpenSRO2*/*SpenSRO3*	1.06	0.79	1.33	Purify selection	segmental	26.48
	Spen5/Spen5	*SpenSRO4*/*SpenSRO5*	0.94	1.36	0.69	Purify selection	segmental	45.33
*S. chilense*	—	*SolcSRO1*/*SolcSRO2*	0.93	1.36	0.69	Purify selection	segmental	45.21
	—	*SolcSRO5*/*SolcSRO6*	0.99	1.05	0.94	Purify selection	segmental	35.05
*S. lycopersicum* var. *cerasiforme*	Slyc1/Slyc1	*SlycSRO1*/*SlycSRO2*	1.01	0.96	1.05	Positive selection	tandem	32.05
	Slyc1/Slyc4	*SlycSRO1*/*SlycSRO4*	0.98	1.09	0.90	Purify selection	segmental	36.40
	Slyc1/Slyc4	*SlycSRO1*/*SlycSRO5*	0.98	1.08	0.90	Purify selection	segmental	36.15
	Slyc1/Slyc4	*SlycSRO1*/*SlycSRO6*	1.05	0.83	1.27	Positive selection	segmental	27.55
	Slyc1/Slyc4	*SlycSRO2*/*SlycSRO4*	1.01	0.96	1.05	Positive selection	segmental	32.12
	Slyc1/Slyc4	*SlycSRO2*/*SlycSRO5*	1.00	1.01	0.99	Purify selection	segmental	33.72
	Slyc1/Slyc4	*SlycSRO2*/*SlycSRO6*	1.02	0.90	1.14	Positive selection	segmental	30.02
	Slyc4/Slyc4	*SlycSRO4*/*SlycSRO6*	1.01	0.97	1.05	Positive selection	segmental	32.19
	Slyc4/Slyc4	*SlycSRO5*/*SlycSRO6*	0.99	1.03	0.96	Purify selection	segmental	34.49
	Slyc4/Slyc6	*SlycSRO5*/*SlycSRO9*	0.96	1.14	0.84	Purify selection	segmental	37.99
	Slyc5/Slyc6	*SlycSRO7*/*SlycSRO8*	0.12	0.45	0.27	Purify selection	tandem	14.86
*S. pimpinellifolium*	Spi1/Spi4	*SpiSRO1*/*SpiSRO3*	1.02	0.91	1.12	Positive selection	segmental	30.39
	Spi1/Spi4	*SpiSRO1*/*SpiSRO4*	1.01	0.97	1.03	Positive selection	segmental	32.48
	Spi4/Spi4	*SpiSRO3*/*SpiSRO4*	0.96	1.16	0.83	Purify selection	tandem	38.52
	Spi4/Spi4	*SpiSRO3*/*SpiSRO5*	0.95	1.19	0.80	Purify selection	segmental	39.68
	Spi5/Spi5	*SpiSRO6*/*SpiSRO7*	0.15	0.17	0.87	Purify selection	tandem	5.62
	Spi6/Spi8	*SpiSRO8*/*SpiSRO10*	0.99	1.06	0.93	Purify selection	segmental	35.30
*S. lycopersicoides*	Slyd5/Slyd8	solydSRO2/solydSRO5	1.00	0.99	1.01	Positive selection	segmental	33.05
	Slyd6/Slyd8	solydSRO3/solydSRO4	0.98	1.09	0.90	Purify selection	segmental	36.21

a Millions years ago.

### Evolutionary and Collinearity Analysis of *SRO* Genes in Tomato

**FIGURE 4:**
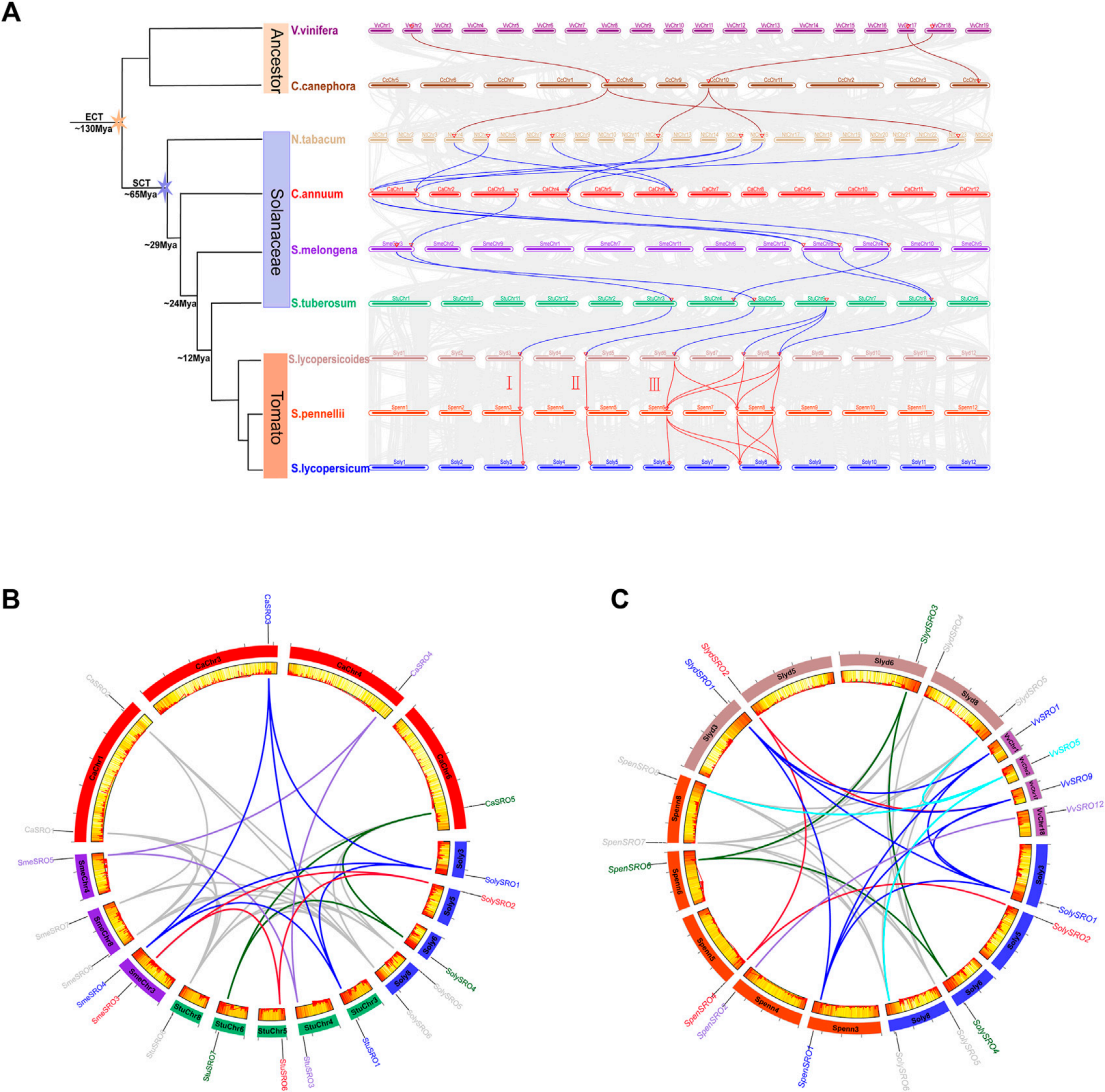
Homologous genes and evolutionary analysis of the SRO family. **(A)** Co-lineage map for multiple species, with species genomes arranged in evolutionary order and coloured lines representing *SRO* genes with direct homologous relationships within each species. **(B)** Co-lineage map of *SRO* genes within *Solanaceae* (*C. annuum*, *S. melongena*, *S. tuberosum*, and *S. lycopersicum*), with the outer circle showing the chromosomes of each *Solanaceae*, the inner circle showing gene density, the ends of the lines representing direct homologous *SRO* genes, and the different coloured lines representing different evolutionary patterns. **(C)** Covariance of *SRO* genes within *V. vinifera.* L, *S. lycopersicoides*, *S. pennellii*, and *S. lycopersicum*, with the outer circle showing the chromosomes of each species, the inner circle showing the gene density, the two ends of the lines representing the direct homologous *SRO* genes, and different coloured lines representing different evolutionary patterns.

To trace the evolutionary origin and orthologous relationship of the *SRO* genes in tomatoes, we used grape (*Vitis vinifera*. L) and coffee (*Coffea canephora*), which did not undergo a new specific genome-wide doubling event after a “gamma” whole-genome triplication event that was common to most ancient ancestors of eudicot plants (Wang et al., 2016). At the same time, according to the time of *Solanaceae* differentiation, the *SRO* genes were analysed for interspecies collinearity (Figure 4A, Supplementary Table S3). The SRO family expanded with the whole genome replication of angiosperms. Grape and coffee, which represent the ancient ancestors, each had three *SRO* genes, which were highly conserved in the evolutionary process and homologous with several *SRO* genes in *Solanaceae*. This indicated that the SRO family in plants may be copied from one *SRO* gene in the ancestral species after the *γ* event. Starting from tobacco, the number of homologous members of the SRO family increased to five, and the evolutionary speed was accelerated. The SRO family in tomato was divided into subfamilies I–III, in which subfamilies I and II each contained only one *SRO* gene on Chr3 and Chr4, respectively. Subfamily III contained three *SRO* genes on Chr6 and Chr8, and *SRO* genes in all subfamilies were highly homologous in both cultivated and wild tomatoes.

To further discover the origin of the SRO family in tomatoes, we extracted the collinearity of the SRO family in *Solanaceae* (*C. annuum*, *S. tuberosum*, and *S. melongena*), V. *vinifera*. L and various tomatoes (*S. lycopersicoides*, *S. pennellii*, and *S. lycopersicum*) (Figures 4B,C). We realized that the *SRO* genes in tomatoes actually showed five orthologous patterns based on the position of its chromosome. *SolySRO1*, located on Chr3, had one orthologous gene in all *Solanaceae* and two orthologous genes (*VvSRO1* and *VvSRO9*) in grape. *SolySRO2*, located on Chr5, had one homologous gene with all other species and only lacked a homologous relationship in *C. annuum*. This gene was also derived from *VvSRO9* and maintained a certain degree of conservation during evolution. *SolySRO4*, located on Chr6, has one homologous gene in all *Solanaceae*, with the exception of *S. melongena*, and there is no homologous *SRO* member in grape. The *SolySRO5* and *SolySRO6* gene pairs located on Chr8 had highly homologous *SRO* genes in all species. Interestingly, there was only one *VvSRO5* homologous gene in grape. We also noticed that *SpenSRO2* on Chr4 in *S. pennellii* is highly homologous to *VvSRO12*. Chr4 in several *Solanaceae* also contained homologous *SRO* genes, which were lost in cultivated tomato.

### Interaction Between Protein and microRNA of *SRO* Genes in Tomato

**FIGURE 5:**
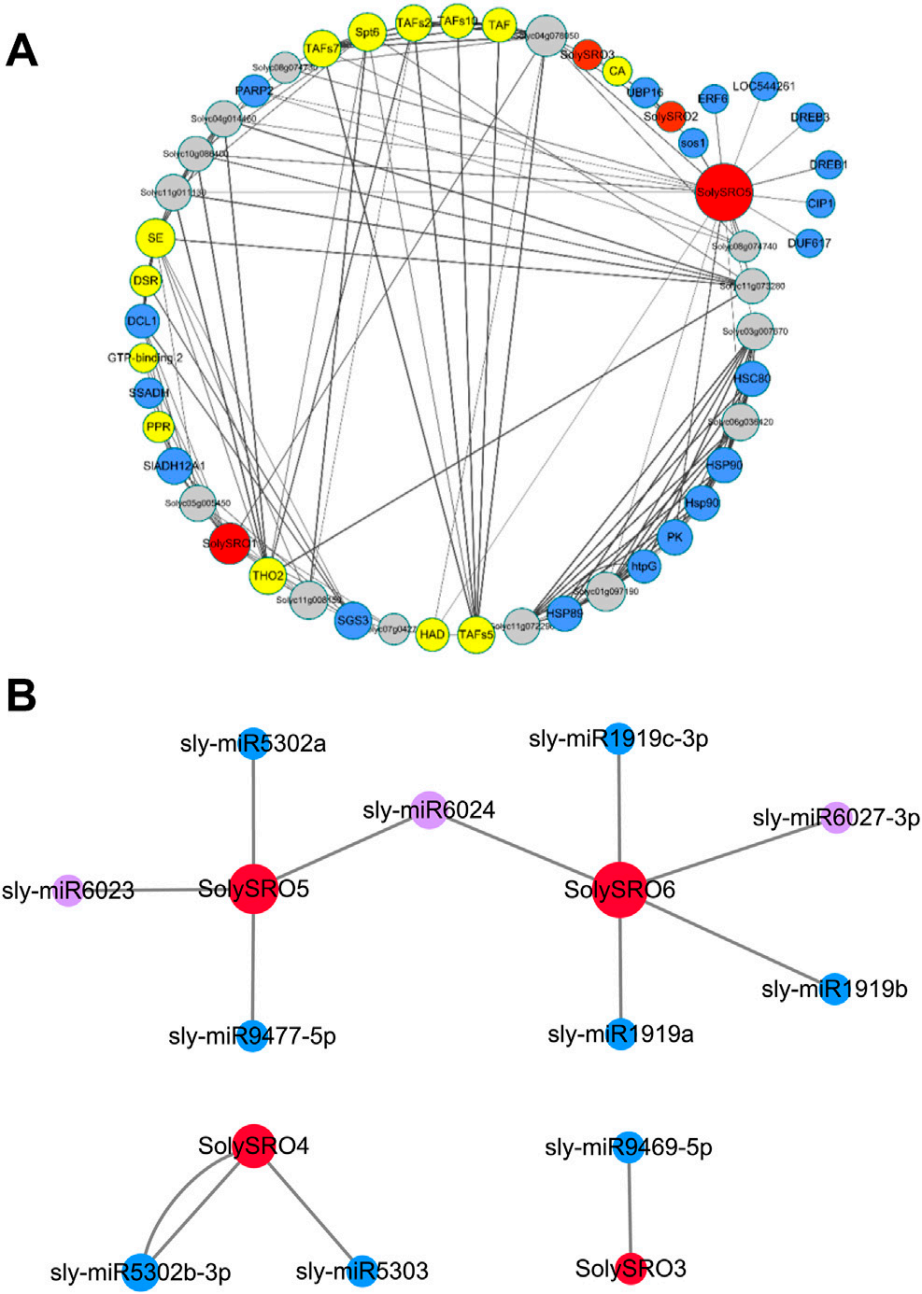
Inter-crossing network diagram of *SRO* genes in tomatoes. **(A)** Interaction network of the SRO family with other proteins. Each node is a protein, each edge represents the presence of interactions, the size of the node represents the number of interactions, the thickness of the edge represents the value of the combined score, red nodes represent SRO proteins, blue nodes represent stress-related proteins, yellow nodes are transcription factors, and grey nodes represent proteins lacking annotation. **(B)** *SRO* genes and micoRNA targeting interactions. The red nodes are *SRO* genes, the size of the node represents the number of interactions, the blue nodes represent abiotic stress-related microRNAs, and the purple nodes are biotic stress-related microRNAs.

To better understand the function of *SRO* genes in tomatoes, we predicted the interactions between all SolySROs proteins based on the STRING online database. SolySRO4 and SolySRO6 had no predicted interactions with any protein. There was no direct interaction between SolySRO1, SolySRO2, SolySRO3, and SolySRO5, but they cooperated with other proteins to regulate similar physiological functions and produced a total of 218 branches (Supplementary Table S4). SolySRO5 interacted with the most proteins with 31, and SolySRO2 and SolySRO3 each interacted with only three proteins. We excluded some proteins with lost annotations and low degree values and drew an interaction network diagram (Figure 5A). The results showed that proteins interacting with the SolySRO family could be divided into three categories. The number of proteins related to environmental stress response was the largest, including the protein families SLADH, SSADH, and LOC that regulate the balance of ROS products, the HSP, SOS, UBP, etc., which promote plant adaptation to low temperature and participate in plant salt and drought stress tolerance, the protein families that enhance plant biotic stress resistance, SGS and DCL, and the DREB, ERF, and AP2, etc., which are regulated and responded to by hormones. SolySROs also interacted with a large number of transcription factor protein families, including SPT, TAF, and DSR that regulate the transcription process, which may be related to their expression patterns under special circumstances. There were also some proteins with missing annotations in the interaction network diagram. They had a clear direct or indirect synergy with SolySRO proteins, but their functions were still unclear.

MicroRNAs have target regulatory relationships with *SolySROs* were predicted in the psRNATarget database (Figure 5B, Supplementary Table S5). Only four genes, *SolySRO3*, *SolySRO4*, *SolySRO5* and *SolySRO6*, were predicted to have a targeted regulatory relationship. *SolySRO6* was targeted by five microRNAs, with the greatest regulation. *SolySRO5* and *SolySRO4* were targeted by four and two microRNAs, respectively, and *SolySRO3* was only regulated by microRNA9469. Almost all microRNAs targeted a single *SolySRO* gene, with only micoRNA6024 targeting and regulating the *SolySRO5* and *SolySRO6* genes at the same time, and micoRNA5302 bound two specific target sites of *SolySRO4*. The above results of the protein interaction network and microRNA targeting regulation provided more possibilities for functional research on *SolySROs*.

### Expression Profile Analysis of *SRO* Genes in Tomato

**FIGURE 6:**
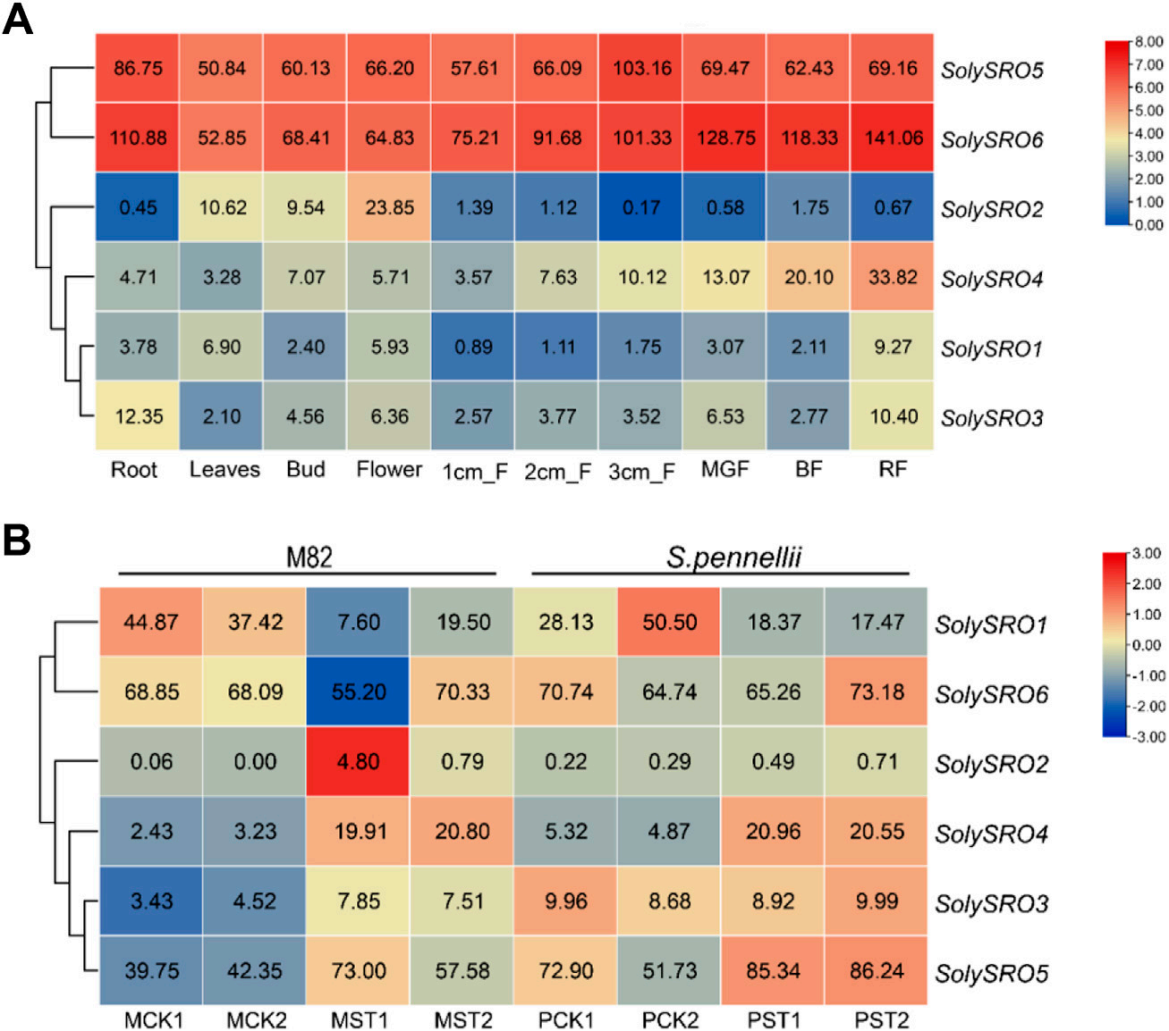
Quantitative heat map of *SRO* gene expression. The color bar represents the log^2^ expression values, With red representing high expression levels and blue representing low expression levels. The gene name is shown on the right side. **(A)** Heat map of tissue-specific expression of *SRO* genes in tomato. Heinz roots (Root), Heinz leaves (Leaves), Heinz unopened flower buds (Bud), Heinz fully opened flowers (Flower), Heinz 1 cm fruits (1 cm_F), Heinz 2 cm fruits (2 cm_F), Heinz 3 cm fruits (3 cm_F), Heinz mature green fruits (MGF), Heinz breaker fruits (BF), Heinz breaker + 10 fruits (RF) **(B)** Heat map of *SRO* gene expression in M82 and *S. pennellii* under salt stress. Normal growth of M82 (MCK), Normal growth of *S. pennellii* (PCK), Salt-stressed M82 (MST), Salt-stressed *S. pennellii* (PST), Each treatment has two replicates.

The published RNA-seq data were used to study the expression pattern of *SolySROs*. The results of *the SRO* genes expression profile in different tomato tissues showed that all *SolySROs* members exhibited strong tissue-specific expression, and they were obviously divided into two groups by expression level (Figure 6A, Supplementary Table S6). *SolySRO5* and *SolySRO6* had higher expression levels in all tomato tissues. The expression level of *SolySRO5* was the highest in fruit (3 cm), and this value of *SolySRO6* appeared in mature fruits, which indicated that these two *SRO* genes were the core genes of the *SRO* family and were highly expressed in fruit development and ripening. *SolySRO1*, *SolySRO2*, *SolySRO3*, and *SolySRO4* were all expressed at low levels in different tissues and were only highly expressed at secific periods. The expression level of *SolySRO2* was highest in flowers. The expression of *SolySRO3* in roots was higher than that in other tissues, while the maximum expression of *SolySRO4* and *SolySRO1* appeared in mature fruits. In addition, based on the RNA-seq data, the expression patterns of *SolySROs* in cultivated tomatoes (M82) and wild tomato (*S. pennellii*) under salt stress were studied (Figure 6B, Supplementary Table S6). The SRO family of cultivated and wild tomatoes exhibited the same expression patterns under a high salt environment. Compared to the control, *SolySRO1* was significantly down-regulated in both M82 (log_2_ FC = 1.39) and *S. pennellii* (log_2_ FC = 1.08). *SolySRO4* was significantly up-regulated in both M82 (log_2_ FC = 2.38) and *S. pennellii* (log_2_ FC = 1.68), which means that *SolySRO4* was the main salt stress response factors in the SRO family. In particular, the expression of *SolySRO2* significantly increased in M82 (log_2_ FC = 6.34) under a salt environment but did not change in *S. pennellii* Although the expression of *SolySRO3* and *SolySRO5* increased, they did not reach the significant level. The expression level of *SolySRO6* remained basically unchanged.

### Expression Profiles of *SolySROs* Under Abiotic Stress and Hormone Treatment

**FIGURE 7:**
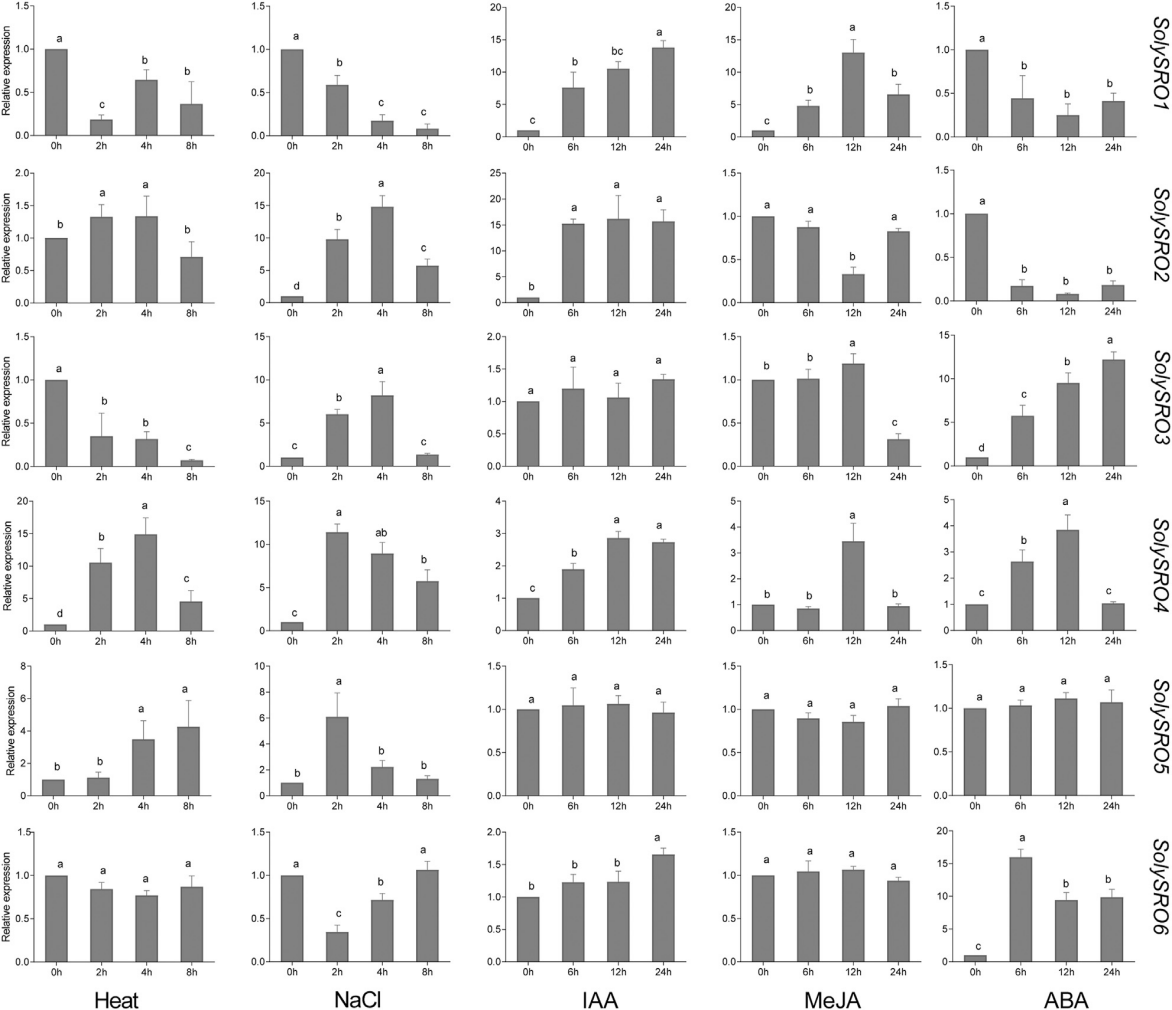
Real-time quantitative PCR validation of *SRO* genes under abiotic stress and hormone induction, The standard deviations are shown with error bars.

To investigate the expression pattern of the *SRO* genes in tomato, qRT-PCR experiments were performed to analyse six *SolySRO* genes under two abiotic stresses and three hormone treatments (Figure 7). Compared to the control, high-temperature stress caused a decrease in the expression of *SolySRO1* (81.60%) and *SolySRO3* (64.99%) in 2 h. The expressions of both *SolySRO2* (32.62%) and *SolySRO4* (953.09%) first increased in 2 h and then decreased by 8 h *SolySRO5* expression continued to increase, and *SolySRO6* expression remained unchanged throughout. The expression pattern of *SRO* under salt stress simulated by NaCl was different. The expression of *SolySRO1* decreased compared to the control, while the expressions of *SolySRO2* (1381.39%) and *SolySRO3* (720.26%) increased and reached a maximum at 4 h. The expressions of *SolySRO4* (1037.57%) and *SolySRO5* (563.12%) also increased, but their maximum expression occurred at 2 h. The expression of *SolySRO6* decreased first and then returned to normal at 8 h. The response of the tomato *SRO* genes was explored with auxin, methyl jasmonate and abscisic acid. The expressions of *SolySRO1*, *SolySRO2*, *SolySRO4*, and *SolySRO6* all increased under the IAA treatment, reaching maximum expression at 12 and 24 h, respectively, and the expressions of *SolySRO3* and *SolySRO5* did not change significantly. The expressions of *SolySRO5* and *SolySRO6* also did not change significantly under the MeJA treatment, while the expressions of *SolySRO1* and *SolySRO4* increased significantly and reached a maximum at 12 h, *SolySRO2* decreased significantly at 12 h and *SolySRO3* was the least expressed at 24 h. Under ABA stress, the expressions of *SolySRO1* and *SolySRO2* decreased throughout and did not recover, while the expressions of *SolySRO3*, *SolySRO4* and *SolySRO6* increased significantly and reached a maximum at 24, 12 and 6 h, respectively, while the expression of *SolySRO5* did not change significantly throughout the stress.

## Discussion

Next-generation sequencing (NGS) technology improves the resolution and accuracy of genomics research, focusing on repeated prediction and verification of a few genes, avoiding the annotation errors of individual gene sequences caused by genome-wide sequencing, and enabling genetic improvement and directional breeding of plants (Rothan et al., 2019). As a small protein family unique to plants, *SRO* has been suggested to participate in a variety of abiotic stress and oxidative stress responses in plant growth, thereby enhancing plant stress tolerance. *SRO* has been isolated and identified in a variety of plants (You et al., 2014; Li et al., 2017; Jiang et al., 2018; Zhang et al., 2019; Jiang et al., 2020). In this study, we systematically identified SRO family members in a variety of tomatoes and studied their physical and chemical properties, structural characteristics, evolutionary classification and functional expression. Like most higher plants, cultivated tomato also contains 6 members of the SRO family. This number is the same as that of Arabidopsis and bananas but less than that of wheat (Ahlfors et al., 2004; Zhang et al., 2019; Jiang et al., 2020). In cultivated tomato, the SRO family is distributed on chromosomes 3, 5, 6 and 8, which was highly consistent with *S. lycopersicoides*but different from the wild tomatoes. The additional *SRO* members in wild tomatoes were mainly distributed on Chr1 and Chr4. The *SRO* genes in tomatoes show a certain degree of conservation and separation along with their distribution on the chromosome. *SRO* genes at the same or similar positions on different tomatoes chromosomes were highly consistent in their physical and chemical properties such as amino acid length, molecular weight, and isoelectric point. Similarly, *SRO* genes distributed on different chromosomes were quite different in both cultivated tomato and wild tomatoes. Based on the conservative characteristics of *SRO* genes in chromosome distribution, we can predict that *SRO* genes in *S. chilense* were also distributed on Chr1 (*SolcSRO6*), Chr3 (*SolcSRO7*), Chr5 (*SolcSRO1* and *SolcSRO2*), Chr6 and Chr8 (*SolcSRO3* and *SolcSRO4*, *SolcSRO5*), even if they were not mounted on chromosomes.

The differential functional expression of genes is closely related to their structures. Similar to the physical and chemical characteristics, whether in cultivated tomato or wild tomatoes, *SRO* genes distributed in the same or similar positions on chromosomes also had similar structures and conserved motifs. The *SRO* genes in cultivated tomato were divided into group I and group II, and group III was added by wild tomatoes. The SRO family in tomato is undoubtedly conserved. The *SRO* genes in the same group showed similar numbers of exons and conserved structures in a variety of tomatoes, especially the *SRO* genes in group I, which had a highly consistent exon distribution and the largest number of motifs and were likely the core gene cluster in the tomato SRO family. However, compared with wild tomatoes, *SRO* genes in cultivated tomato often have longer gene structures and more introns than other genes in the same group, which means that *SolySROs* can achieve transcriptional diversification through alternative splicing and other processes, thus regulating more complex and extensive functions (Liu et al., 2021). This was obviously not available in the *SRO* gene in wild tomatoes. We speculated that artificial domestication may cause the loss of *SolySROs* genes on Chr1 and Chr4. Mutations may also increase the complexity of the *SolySROs* gene structure, thereby maintaining the functional expression of the SRO family and reducing gene redundancy. Unfortunately, we have not found similar reports in SRO family studies of other species.

Predicting the promoter sequence of *SRO* genes in tomato, we found 87 CREs, it indicated that the SRO family was widely involved in mediating multiple life activities of tomato. The distribution of hormone response elements was the most widespread. Both cultivated and wild tomatoes *SRO* genes contained a large number of response elements, including gibberellin, ethylene, abscisic acid, jasmonic acid, and salicylic acid. *SRO* genes may affect tomato life activities by widely participating in hormone regulation networks, which is consistent with studies in other species (YongChun et al., 2019; Qiao et al., 2020). There were also a large number of light-responsive CREs in the *SRO* promoter region, mainly BOX-4 and G-BOX, and most light-responsive elements were significantly enriched in *S. lycopersicum* var. *cerasiforme* and *S. pimpinellifolium*, which was consistent with their light-loving and heat-resistant growth characteristics (Kumar et al., 2015). Stress-related response elements showed that many *SRO* genes were induced by an anaerobic response. The stress-related elements of SRO family members in cultivated tomato were far less abundant than those in wild tomatoes, which may lead to damage to their stress tolerance.

With the different evolutionary statuses of the plants, there were obvious differences in the *SRO* genes. The phylogenetic tree showed that genome evolution of the SRO family followed the differentiation of species, Bryophytes, Tracheophytes, Monocots and Eudicots were distributed in different branches. it was consistent with some previous studies (Zhang et al., 2019; Jiang et al., 2020). *P. patens* and *S. moellendorffii*, which have relatively simple life structures, naturally contained only a few *SRO* genes. With the occurrence of genome-wide replication events (WGD), the number of *SRO* genes gradually increases in some monocotyledons and dicotyledons, indicated that *SRO* genes did undergo lineage-specific amplification and evolution with plant differentiation. According to the phylogenetic tree of *Solanaceae*, the SRO family is more accurately divided into three subgroups. The *SRO* genes structure and typical domains in group I were relatively complete, while the *SRO* genes in groups II and III were either short in length or contained only one of the conserved RST or PARP domains. The *SRO* genes are relatively conserved in *Solanaceae*, and the genetic relationship could not be strictly divided. *SRO* genes in different *Solanaceae* may perform similar functions. Group III contained *C. annuum*, *S. tuberosum*, *S. melongena* and wild tomatoes, but cultivated tomatoes were lost from this group. Long-term artificial domestication caused the SRO family in tomato to shrink.

The proportion of nonsynonymous substitutions (*KAs*) and synonymous substitutions (*KSs*) reflects the selection pressure of gene evolution to a certain extent, generally believed that *Ka*/*Ks* > 1 represents positive selection of accelerated evolution and *Ka*/*Ks* < 1 exhibits gene duplication suffers purifying selection (Wang et al., 2010). The *Ka*/*Ks* ratio of all duplicated wheat SRO gene pairs were <1 (Jiang et al., 2020), The *Ka*/*Ks* ratio of both homologous gene pairs in cultivated tomatoes was also less than 1, these duplicated gene pairs were subject to greater selective pressure and did not produce significant functional differences during evolution. Interestingly, although The *Ka*/*Ks* ratio of most duplicated gene pairs were <1 in wild tomato, there were still a considerable number of duplicated genes *Ka*/*Ks* > 1, and some of them were from tandem repeats, implying that they were subject to environmental positive selection and still in a rapid evolutionary stage. We speculate that the more complex survival environment has forced wild tomatoes to retain the viability of some adaptive genes (Pailles et al., 2017; Gibson and Moyle, 2020) The evolution of genes in the same family often reflects certain key events in the process of species differentiation and maps the source of conservation and differential functions of its family members. Multispecies orthologous genes showed the complete evolutionary trajectory of the SRO family in tomato. The ancient ancestors of angiosperms contained only one *SRO* gene, and duplicated with the occurrence of WGT-*γ*. Approximately 65 Mya, The occurrence of *Solanaceae* exclusive polyploidization event drived massive expansion of SRO genes, the number of family members gradually increased, and the evolution speed accelerated. Approximately 12 Mya, with potato and tomato began to separate, the evolution of the SRO family slowed. *SolySRO1* is the most conserved member in *Solanaceae*. derived from the loss or degeneracy of two ancestral *SRO* genes after triploidization, *SolySRO2* also maintained a certain degree of similarity between the ancestral species. It formed *SolySRO3* through segmental duplication. *SolySRO4* only had orthologous genes in *Solanaceae* and no homologous relationship with grape. This meant that the *SRO* genes of Chr6 may only exists exclusively in *Solanaceae*. *SolySRO5* and *SolySRO6* have two highly homologous colinearity gene pairs in all *Solanaceae*, while only *VvSRO5* had homology with *SolySRO6* in grape. We suggested that an *SRO* genes that was triploidized in the ancestral species replicated in the genome-wide doubling event peculiar to the differentiation stage of *Solanaceae* and preserved in the evolutionary process, formed two members, *SolySRO5* and *SolySRO6*, and then tomato Chr6 and Chr8 underwent gene exchange to form *SolySRO4*. The *SRO* genes deleted on Chr4 in cultivated tomato had orthologous genes in both *Solanaceae* and grape, which further proved that the diversity of the SRO family in cultivated tomato was reduced by domestication.

The prediction results for SRO protein interactions in tomato showed that the SolySRO protein is widely involved in a variety of stress-related pathways. Among them, SLADH and SSADH respond to O_3_ stress and encode aldehyde dehydrogenase to catalyse the conversion of ROS products (Sunkar et al., 2003; Timpson et al., 2012), LOC belongs to the glutathione peroxidase family, which catalyse the reduction of H_2_O_2_ or other organic hydroperoxides in to water or the corresponding alcohols (Islam et al., 2017), The heat shock protein family could significantly promote the ability of tomato to adapt to temperature (Hossain and Nakamoto, 2002), Overexpression of the *SOS* gene significantly improved the salt tolerance of *Arabidopsis thaliana* (Yang et al., 2009), Ubp 16 could interact with specific proteins to improve the tolerance of plants to the heavy metal cadmium (Zhao et al., 2013), The synergistic expression of SolySROs with these proteins undoubtedly improves the ability of tomato to withstand adverse environmental stresses. In Arabidopsis, *AtSRO5* mediates the formation of 24-nt-siRNA by biogenesis pathways such as DCL2 and SGS3 to accumulate proline and improve salt tolerance, while *AtSRO5* similarly reduces ROS products (Mourrain et al., 2000; Borsani et al., 2005; Deleris et al., 2006). Amazing, SolySRO1 is predicted to interact with DCL1 and SGS3 proteins, which may suggest that SolySRO1 mediates tomato proline metabolic synthesis and ROS homeostatic balance through a similar regulatory mode as Arabidopsis AtSRO5-siRNA. Six TaSROs proteins in wheat were predicted to interact with 14 transcription factors (Jiang et al., 2020). SolySROs also interacted with a large number of TFs and the RST domain always acts as the binding sites. This domain may be required for the interaction and co-expression of *SRO* genes with TFs to participate in plant stress resistance in tomato.

Poly (ADP-ribose) polymerase (PARP) widely mediates plant DNA repair, epigenetics and transcription by modifying (poly (ADP-ribosyl) ates) itself and other nuclear proteins (Vainonen et al., 2016; Briggs et al., 2017). Pharmacological inhibition assays suggest that PARP protein is involved in the natural immunity of plants against microorganisms (Adams- Phillips et al., 2009). However, the parp triple mutant whcih knocked out all three *Arabidopsis thaliana PARP* genes did not differ from wild type. Previous research hypothesized that the PARP-like structural domain of the *SRO* gene could serve as an alternative pathway when PARP activity is genetically reduced, even though the domain in the *SRO* gene did not possess any enzymatic activity and its protein sequence was similarly less similar to PARP proteins (Lamb et al., 2012; Rissel et al., 2017). Our study showed that SolySRO5 did have a direct interaction with PARP2 protein, which is the core member of the PARP family in plants (Song et al., 2015), It supported the possibility that *SRO* genes regulated active PARP proteins under specific conditions. Meanwhile *SolySRO5* and *SolysSRO6* were predicted to interact with sly-miR6023, sly-miR6024 and sly-miR6027-3p, these miRNAs regarded to be involved in plant-pathogen interactions and could regulate R gene expression in tomato (Prigigallo et al., 2019), and all four miRNA targeting sites were located in the PARP-like domain of *SRO* genes, suggesting the complexity of the active PARP protein being regulated by SRO genes. We know little about the involvement of the SRO family in plant biological stress, Four *MaSROs* showed significant dysregulation of expression in banana roots inoculated with *Fusarium oxysporum* f. sp. *Cubense* (Zhang et al., 2019), Transcriptomic data revealed that *TaSRO1b.3-4A* and *TaSRO2b.3-4B* genes in wheat were responsive to multiple fungal diseases (Jiang et al., 2020). MicroRNA family predicted in our study was conserved in Solanaceae and highly expressed in tomato leaves infected by potato virus (Li et al., 2012; Miozzi et al., 2014), these results likewise provided new insights into the involvement of *SRO* genes in biotic stresses. considering the conservation of SolySRO5 and SolySRO6 in the evolutionary process, we believed that the pattern of miRNA-SRO involvement in plant biotic stress response was at least conserved in *Solanaceae*.

Tissue-specific expression showed that the expression pattern of the SRO family members in tomato was significantly different from that in other plants. *SolySRO1* maintained low expression throughout the reproductive period. *SolySRO2* was highly expressed in seeds and flowers. *SolySRO3* had its highest expression level in roots. This gene may be related to tomato perception and response to stimuli. *SolySRO4* was highly expressed in mature tomato fruits and may be involved in the transformation of green tomato fruit to red fruit by regulating hormones such as ethylene. Compared with other *SolySRO* genes, *SolySRO5* and *SolySRO6* maintained absolute high expression throughout the growth period of tomato. These two genes were widely involved in the dynamics of tomato growth and development and reached maximum expression in the fruit. Salt stress caused an imbalance in SRO family expression, and the expressions of *SolySRO4* increased significantly to cope with the high-salt environment. In this study, the expressions of *SolySROs* under different stress environments were also verified using qRT-PCR. The expressions of *SolySRO2*, *SolySRO4*, and *SolySRO5* significantly increased under both high temperature and salt stress, and these three genes were likely to be more sensitive to the stress response and expressed rapidly in tomato in response to adverse conditions. The expression of *SolySRO4* was significantly increased at 6 h under the IAA, MeJA and ABA treatments after exogenous application of hormones, whereas the expression of *SolySRO5* did not change significantly under the three hormone environments; they all had many hormone-responsive elements distributed in their promoter regions, but the hormone response mechanisms were different. *SolySRO4*, *SolySRO5* and *SolySRO6* were evolutionarily homologous and highly similar in gene structure and conserved motifs, but their expression patterns were not identical. *SolySRO1*, *SolySRO2* and *SolySRO3*, which were distributed in the same subclade, were also highly divergent. The results that *SolySROs* expression patterns did not substantially vary in a simple linear fashion with time, and indeeded in other species (Ahlfors et al., 2004; Zhang et al., 2019; Jiang et al., 2020), provided evidence for the complex expression patterns of *SRO*.

## Conclusion

In this study, we systematically identified the SRO family from the tomato genome and its wild relatives. We used bioinformatics method to describe the physical and chemical properties, gene structure, protein interactions, promoter elements and targeted microRNA regulation of different *SRO* genes. The evolutionary origin of the *SRO* genes in tomato was also discussed. Transcriptome analysis showed that only two genes, *SolySRO5* and *SolySRO6*, were highly expressed in different tissues of tomato and affected and regulated the dynamic changes of tomato development. Four *SolySROs* genes responded significantly to salt stress, of which *SolySRO4* and *SolySRO5* were the core genes. At the same time, the *SRO* genes were verified by qRT-PCR. These genes were involved in hormone-mediated pathways and played an important role in tomato resistance to abiotic stress. These results laid a foundation for further study of the function of the SRO family in tomato and had value for applications in tomato resistance breeding.

## Data Availability

The original contributions presented in the study are included in the article/Supplementary Material, further inquiries can be directed to the corresponding author.

## References

[B1] Adams-Phillips L. Briggs A. G. Bent A. F. (2009). Disruption of Poly(ADP-Ribosyl)ation Mechanisms Alters Responses of Arabidopsis to Biotic Stress. Plant Physiol. 152, 267–280. 10.1104/pp.109.148049 19889874 PMC2799362

[B2] Ahlfors R. Lång S. Overmyer K. Jaspers P. Brosché M. Tauriainen A. (2004). Arabidopsis RADICAL-INDUCED CELL DEATH1 Belongs to the WWE Protein-Protein Interaction Domain Protein Family and Modulates Abscisic Acid, Ethylene, and Methyl Jasmonate Responses. Plant Cell 16, 1925–1937. 10.1105/tpc.021832 15208394 PMC514171

[B3] Artimo P. Jonnalagedda M. Arnold K. Baratin D. Csardi G. de Castro E. (2012). ExPASy: SIB Bioinformatics Resource Portal. Nucleic Acids Res. 40, W597–W603. 10.1093/nar/gks400 22661580 PMC3394269

[B4] Babajani G. Effendy J. Plant A. L. (2009). Sl-SROl1 Increases Salt Tolerance and Is a Member of the Radical-Induced Cell Death 1-similar to RCD1 Gene Family of Tomato. Plant Sci. 176, 214–222. 10.1016/j.plantsci.2008.10.012

[B5] Bateman A. Coin L. Durbin R. Finn R. D. Hollich V. Griffiths‐Jones S. (2004). The Pfam Protein Families Database. Nucleic Acids Res. 32, 138D–141D. 10.1093/nar/gkh121 PMC308855 14681378

[B6] Biłas R. Szafran K. Hnatuszko-Konka K. Kononowicz A. K. (2016). *Cis*-regulatory Elements Used to Control Gene Expression in Plants. Plant Cel Tiss Organ. Cult 127, 269–287. 10.1007/s11240-016-1057-7

[B7] Blanc G. Wolfe K. H. (2004). Widespread Paleopolyploidy in Model Plant Species Inferred from Age Distributions of Duplicate Genes[W]. Plant Cell 16, 1667–1678. 10.1105/tpc.021345 15208399 PMC514152

[B8] Borsani O. Zhu J. Verslues P. E. Sunkar R. Zhu J.-K. (2005). Endogenous siRNAs Derived from a Pair of Natural *Cis*-Antisense Transcripts Regulate Salt Tolerance in Arabidopsis. Cell 123, 1279–1291. 10.1016/j.cell.2005.11.035 16377568 PMC3137516

[B9] Briggs A. G. Adams-Phillips L. C. Keppler B. D. Zebell S. G. Arend K. C. Apfelbaum A. A. (2017). A Transcriptomics Approach Uncovers Novel Roles for Poly(ADP-Ribosyl)ation in the Basal Defense Response in *Arabidopsis thaliana* . Plos One 12, e0190268. 10.1371/journal.pone.0190268 29284022 PMC5746271

[B10] Chen C. Chen H. Zhang Y. Thomas H. R. Frank M. H. He Y. (2020). TBtools: an Integrative Toolkit Developed for Interactive Analyses of Big Biological Data. Mol. Plant 13, 1194–1202. 10.1016/j.molp.2020.06.009 32585190

[B11] Dai X. Zhuang Z. Zhao P. X. (2018). psRNATarget: a Plant Small RNA Target Analysis Server (2017 Release). Nucleic Acids Res. 46, W49–W54. 10.1093/nar/gky316 29718424 PMC6030838

[B12] Deleris A. Gallego-Bartolome J. Bao J. Kasschau K. D. Carrington J. C. Voinnet O. (2006). Hierarchical Action and Inhibition of Plant Dicer-like Proteins in Antiviral Defense. Science 313, 68–71. 10.1126/science.1128214 16741077

[B13] Du H. Zhang L. Liu L. Tang X.-F. Yang W.-J. Wu Y.-M. (2009). Biochemical and Molecular Characterization of Plant MYB Transcription Factor Family. Biochem. Mosc. 74, 1–11. 10.1134/S0006297909010015 19232042

[B14] Fei Z. Joung J.-G. Tang X. Zheng Y. Huang M. Lee J. M. (2011). Tomato Functional Genomics Database: a Comprehensive Resource and Analysis Package for Tomato Functional Genomics. Nucleic Acids Res. 39, D1156–D1163. 10.1093/nar/gkq991 20965973 PMC3013811

[B15] Fernandez-Pozo N. Menda N. Edwards J. D. Saha S. Tecle I. Y. Strickler S. R. (2015). The Sol Genomics Network (SGN)-from Genotype to Phenotype to Breeding. Nucleic Acids Res. 43, D1036–D1041. 10.1093/nar/gku1195 25428362 PMC4383978

[B16] Finn R. D. Clements J. Eddy S. R. (2011). HMMER Web Server: Interactive Sequence Similarity Searching. Nucleic Acids Res. 39, W29–W37. 10.1093/nar/gkr367 21593126 PMC3125773

[B17] Gibson M. J. S. Moyle L. C. (2020). Regional Differences in the Abiotic Environment Contribute to Genomic Divergence within a Wild Tomato Species. Mol. Ecol. 29, 2204–2217. 10.1111/mec.15477 32419208

[B18] Goodstein D. M. Shu S. Howson R. Neupane R. Hayes R. D. Fazo J. (2012). Phytozome: a Comparative Platform for green Plant Genomics. Nucleic Acids Res. 40, D1178–D1186. 10.1093/nar/gkr944 22110026 PMC3245001

[B19] Grundy W. N. Bailey T. L. Elkan C. P. Baker M. E. (1997). Meta-MEME: Motif-Based Hidden Markov Models of Protein Families. Bioinformatics 13, 397–406. 10.1093/bioinformatics/13.4.397 9283754

[B20] Horton P. Park K.-J. Obayashi T. Fujita N. Harada H. Adams-Collier C. J. (2007). WoLF PSORT: Protein Localization Predictor. Nucleic Acids Res. 35, W585–W587. 10.1093/nar/gkm259 17517783 PMC1933216

[B21] Hossain M. M. Nakamoto H. (2002). HtpG Plays a Role in Cold Acclimation in Cyanobacteria. Curr. Microbiol. 44, 291–296. 10.1007/s00284-001-0005-9 11910501

[B22] Hu B. Jin J. Guo A.-Y. Zhang H. Luo J. Gao G. (2015). GSDS 2.0: an Upgraded Gene Feature Visualization Server. Bioinformatics 31, 1296–1297. 10.1093/bioinformatics/btu817 25504850 PMC4393523

[B23] Islam S. Rahman I. A. Islam T. Ghosh A. (2017). Genome-wide Identification and Expression Analysis of Glutathione S-Transferase Gene Family in Tomato: Gaining an Insight to Their Physiological and Stress-specific Roles. PLoS One 12, e0187504. 10.1371/journal.pone.0187504 29095889 PMC5667761

[B24] Jaspers P. Blomster T. Brosché M. Salojärvi J. Ahlfors R. Vainonen J. P. (2009). Unequally Redundant *RCD1* and *SRO1* Mediate Stress and Developmental Responses and Interact with Transcription Factors. Plant J. 60, 268–279. 10.1111/j.1365-313X.2009.03951.x 19548978

[B25] Jaspers P. Brosché M. Overmyer K. Kangasjär J. (2010a). The Transcription Factor Interacting Protein *RCD1* Contains a Novel Conserved Domain. Plant Signaling Behav. 5, 78–80. 10.4161/psb.5.1.10293 PMC2835967 20592818

[B26] Jaspers P. Overmyer K. Wrzaczek M. Vainonen J. P. Blomster T. Salojärvi J. (2010b). The RST and PARP-like Domain Containing SRO Protein Family: Analysis of Protein Structure, Function and Conservation in Land Plants. BMC Genomics 11, 170. 10.1186/1471-2164-11-170 20226034 PMC2848248

[B27] Jiang H. Xiao Y. Zhu S. (2018). Genome-wide Identification, Systematic Analysis and Characterization of *SRO* Family Genes in maize (Zea mays L.) Acta Physiol. Plant 40, 176. 10.1007/s11738-018-2738-0

[B28] Jiang W. Geng Y. Liu Y. Chen S. Cao S. Li W. (2020). Genome-wide Identification and Characterization of *SRO* Gene Family in Wheat: Molecular Evolution and Expression Profiles during Different Stresses. Plant Physiol. Biochem. 154, 590–611. 10.1016/j.plaphy.2020.07.006 32912491

[B29] Krishna R. Karkute S. G. Ansari W. A. Jaiswal D. K. Verma J. P. Singh M. (2019). Transgenic Tomatoes for Abiotic Stress Tolerance: Status and Way Ahead. 3 Biotech. 9, 143. 10.1007/s13205-019-1665-0 PMC6423223 30944790

[B30] Kumar P. P. Longjam M. Sikder S. (2015). Morphological Characterisation of Tomato Wild Relatives. Jrnl. Func. Env. Bot. 5, 141. 10.5958/2231-1750.2015.00020.7

[B31] Kumar S. Stecher G. Tamura K. (2016). MEGA7: Molecular Evolutionary Genetics Analysis Version 7.0 for Bigger Datasets. Mol. Biol. Evol. 33, 1870–1874. 10.1093/molbev/msw054 27004904 PMC8210823

[B32] Lamb R. S. Citarelli M. Teotia S. (2012). Functions of the poly(ADP-Ribose) Polymerase Superfamily in Plants. Cell. Mol. Life Sci. 69, 175–189. 10.1007/s00018-011-0793-4 21861184 PMC11114847

[B33] Li F. Pignatta D. Bendix C. Brunkard J. O. Cohn M. M. Tung J. (2012). MicroRNA Regulation of Plant Innate Immune Receptors. Proc. Natl. Acad. Sci. 109, 1790–1795. 10.1073/pnas.1118282109 22307647 PMC3277104

[B34] Li B.-Z. Zhao X. Zhao X.-L. Peng L. (2013). Structure and Function Analysis of *Arabidopsis thaliana* SRO Protein Family. Hereditas (Beijing) 35, 1189–1197. 10.3724/SP.J.1005.2013.01189 24459892

[B35] Li H. Li R. Qu F. Yao J. Hao Y. Wang X. (2017). Identification of the SRO Gene Family in Apples (Malus×domestica) with a Functional Characterization of MdRCD1. Tree Genet. Genomes 13, 94. 10.1007/s11295-017-1175-3

[B36] Li Z. Shen J. Liang J. (2019). Genome-Wide Identification, Expression Profile, and Alternative Splicing Analysis of the Brassinosteroid-Signaling Kinase (BSK) Family Genes in Arabidopsis. Ijms 20, 1138. 10.3390/ijms20051138 PMC6429265 30845672

[B37] Li W. Pang S. Lu Z. Jin B. (2020). Function and Mechanism of WRKY Transcription Factors in Abiotic Stress Responses of Plants. Plants 9, 1515. 10.3390/plants9111515 PMC7695288 33171689

[B38] Lin T. Zhu G. Zhang J. Xu X. Yu Q. Zheng Z. (2014). Genomic Analyses Provide Insights into the History of Tomato Breeding. Nat. Genet. 46, 1220–1226. 10.1038/ng.3117 25305757

[B39] Liu H. Lyu H. M. Zhu K. Van de Peer Y. Cheng Z. M. Max (2021). The Emergence and Evolution of Intron‐poor and Intronless Genes in Intron‐rich Plant Gene Families. Plant J. 105, 1072–1082. 10.1111/tpj.15088 33217085 PMC7116809

[B40] Livak K. J. Schmittgen T. D. (2001). Analysis of Relative Gene Expression Data Using Real-Time Quantitative PCR and the 2−ΔΔCT Method. Methods 25, 402–408. 10.1006/meth.2001.1262 11846609

[B41] Love M. I. Huber W. Anders S. (2014). Moderated Estimation of Fold Change and Dispersion for RNA-Seq Data with DESeq2. Genome Biol. 15, 550. 10.1186/s13059-014-0550-8 25516281 PMC4302049

[B42] Marchler-Bauer A. Anderson J. B. Derbyshire M. K. DeWeese-Scott C. Gonzales N. R. Gwadz M. (2007). CDD: A Conserved Domain Database for Interactive Domain Family Analysis. Nucleic Acids Res. 35, D237–D240. 10.1093/nar/gkl951 17135202 PMC1751546

[B43] Miozzi L. Napoli C. Sardo L. Accotto G. P. (2014). Transcriptomics of the Interaction between the Monopartite Phloem-Limited Geminivirus Tomato Yellow Leaf Curl Sardinia Virus and *Solanum lycopersicum* Highlights a Role for Plant Hormones, Autophagy and Plant Immune System Fine Tuning during Infection. PLoS One 9, e89951. 10.1371/journal.pone.0089951 24587146 PMC3938563

[B44] Mourrain P. Béclin C. Elmayan T. Feuerbach F. Godon C. Morel J.-B. (2000). Arabidopsis *SGS2* and *SGS3* Genes Are Required for Posttranscriptional Gene Silencing and Natural Virus Resistance. Cell 101, 533–542. 10.1016/S0092-8674(00)80863-6 10850495

[B45] Nevo E. (2001). Evolution of Genome-Phenome Diversity under Environmental Stress. Proc. Natl. Acad. Sci. 98, 6233–6240. 10.1073/pnas.101109298 11371642 PMC33451

[B46] Overmyer K. Tuominen H. Kettunen R. Betz C. Langebartels C. Sandermann H. Jr. (2000). Ozone-Sensitive Arabidopsis *Rcd1* Mutant Reveals Opposite Roles for Ethylene and Jasmonate Signaling Pathways in Regulating Superoxide-dependent Cell Death. Plant Cell 12, 1849–1862. 10.1105/tpc.12.10.1849 11041881 PMC149124

[B47] Pailles Y. Ho S. Pires I. S. Tester M. Negrão S. Schmöckel S. M. (2017). Genetic Diversity and Population Structure of Two Tomato Species from the Galapagos Islands. Front. Plant Sci. 8, 138. 10.3389/fpls.2017.00138 28261227 PMC5309213

[B48] Prigigallo M. I. Križnik M. De Paola D. Catalano D. Gruden K. Finetti-Sialer M. M. (2019). Potato Virus Y Infection Alters Small RNA Metabolism and Immune Response in Tomato. Viruses 11, 1100. 10.3390/v11121100 PMC6950276 31783643

[B49] Qiao Y. Gao X. Liu Z. Wu Y. Hu L. Yu J. (2020). Genome-Wide Identification and Analysis of SRO Gene Family in Chinese Cabbage (Brassica Rapa L). Plants 9, 1235. 10.3390/plants9091235 PMC7569827 32962109

[B50] Rhee S. Y. Beavis W. Berardini T. Z. Chen G. Dixon D. Doyle A. (2003). The Arabidopsis Information Resource (TAIR): A Model Organism Database Providing a Centralized, Curated Gateway to Arabidopsis Biology, Research Materials and Community. Nucleic Acids Res. 31, 224–228. 10.1093/nar/gkg076 12519987 PMC165523

[B51] Rissel D. Heym P. P. Thor K. Brandt W. Wessjohann L. A. Peiter E. (2017). No Silver Bullet - Canonical Poly(ADP-Ribose) Polymerases (PARPs) Are No Universal Factors of Abiotic and Biotic Stress Resistance of *Arabidopsis thaliana* . Front. Plant Sci. 08, 59. 10.3389/fpls.2017.00059 PMC5292411 28220129

[B52] Rombauts S. Déhais P. Van Montagu M. Rouzé P. (1999). PlantCARE, a Plant *Cis*-Acting Regulatory Element Database. Nucleic Acids Res. 27, 295–296. 10.1093/nar/27.1.295 9847207 PMC148162

[B53] Rothan C. Diouf I. Causse M. (2019). Trait Discovery and Editing in Tomato. Plant J. 97, 73–90. 10.1111/tpj.14152 30417464

[B54] Schultz J. Copley R. R. Doerks T. Ponting C. P. Bork P. (2000). SMART: A Web-Based Tool for the Study of Genetically Mobile Domains. Nucleic Acids Res. 28, 231–234. 10.1093/nar/28.1.231 10592234 PMC102444

[B55] Song J. Keppler B. D. Wise R. R. Bent A. F. (2015). PARP2 Is the Predominant Poly(ADP-Ribose) Polymerase in Arabidopsis DNA Damage and Immune Responses. Plos Genet. 11, e1005200. 10.1371/journal.pgen.1005200 25950582 PMC4423837

[B56] Song L. Huang S.-s. C. Wise A. Castanon R. Nery J. R. Chen H. (2016). A Transcription Factor Hierarchy Defines an Environmental Stress Response Network. Science 354, aag1550. 10.1126/science.aag1550 27811239 PMC5217750

[B57] Sonnhammer E. L. L. Eddy S. R. Durbin R. (1997). Pfam: A Comprehensive Database of Protein Domain Families Based on Seed Alignments. Proteins 28, 405–420. 10.1002/(sici)1097-0134(199707)28:3<405:aid-prot10>3.0.co;2-l 9223186

[B58] Su G. Morris J. H. Demchak B. Bader G. D. (2014). Biological Network Exploration with Cytoscape 3. Curr. Protoc. Bioinf. 47, 8.13.1–8.13.24. 10.1002/0471250953.bi0813s47 PMC4174321 25199793

[B59] Sun X. Wang Y. Sui N. (2018). Transcriptional Regulation of *bHLH* During Plant Response to Stress. Biochem. Biophys. Res. Commun. 503, 397–401. 10.1016/j.bbrc.2018.07.123 30057319

[B60] Sunkar R. Bartels D. Kirch H.-H. (2003). Overexpression of a Stress-Inducible Aldehyde Dehydrogenase Gene from *Arabidopsis thaliana* in Transgenic Plants Improves Stress Tolerance. Plant J. 35, 452–464. 10.1046/j.1365-313X.2003.01819.x 12904208

[B62] Szklarczyk D. Gable A. L. Lyon D. Junge A. Wyder S. Huerta-Cepas J. (2019). STRING V11: Protein-Protein Association Networks with Increased Coverage, Supporting Functional Discovery in Genome-Wide Experimental Datasets. Nucleic Acids Res. 47, D607–D613. 10.1093/nar/gky1131 30476243 PMC6323986

[B63] Szymański J. Bocobza S. Panda S. Sonawane P. Cárdenas P. D. Lashbrooke J. (2020). Analysis of Wild Tomato Introgression Lines Elucidates the Genetic Basis of Transcriptome and Metabolome Variation Underlying Fruit Traits and Pathogen Response. Nat. Genet. 52, 1111–1121. 10.1038/s41588-020-0690-6 32989321

[B64] Teotia S. Lamb R. S. (2009). The Paralogous Genes RADICAL-INDUCED CELL DEATH1 and SIMILAR TO RCD ONE1 Have Partially Redundant Functions during Arabidopsis Development. Plant Physiol. 151, 180–198. 10.1104/pp.109.142786 19625634 PMC2736012

[B65] Teotia S. Lamb R. S. (2011). *RCD1* and *SRO1* Are Necessary to Maintain Meristematic Fate in *Arabidopsis thaliana* . J. Exp. Bot. 62, 1271–1284. 10.1093/jxb/erq363 21172813 PMC3022410

[B66] Timpson L. M. Alsafadi D. Mac Donnchadha C. Liddell S. Sharkey M. A. Paradisi F. (2012). Characterization of Alcohol Dehydrogenase (*ADH12*) from Haloarcula Marismortui, an Extreme Halophile from the Dead Sea. Extremophiles 16, 57–66. 10.1007/s00792-011-0405-0 22015539

[B67] Vainonen J. P. Shapiguzov A. Vaattovaara A. Kangasjärvi J. (2016). Plant PARPs, PARGs and PARP-like Proteins. Curr. Protein Pept. Sci. 17, 713–723. 10.2174/1389203717666160419144721 27090905

[B68] Wang D. Zhang Y. Zhang Z. Zhu J. Yu J. (2010). KaKs_Calculator 2.0: a Toolkit Incorporating Gamma-Series Methods and Sliding Window Strategies. Genomics, Proteomics Bioinf. 8, 77–80. 10.1016/S1672-0229(10)60008-3 PMC5054116 20451164

[B69] Wang Y. Tang H. DeBarry J. D. Tan X. Li J. Wang X. (2012). MCScanX: a Toolkit for Detection and Evolutionary Analysis of Gene Synteny and Collinearity. Nucleic Acids Res. 40, e49. 10.1093/nar/gkr1293 22217600 PMC3326336

[B70] Wang X. Guo H. Wang J. Lei T. Liu T. Wang Z. (2016). Comparative Genomic De‐convolution of the Cotton Genome Revealed a Decaploid Ancestor and Widespread Chromosomal Fractionation. New Phytol. 209, 1252–1263. 10.1111/nph.13689 26756535

[B71] Xu G. Guo C. Shan H. Kong H. (2012). Divergence of Duplicate Genes in Exon-Intron Structure. Proc. Natl. Acad. Sci. 109, 1187–1192. 10.1073/pnas.1109047109 22232673 PMC3268293

[B72] Yang Q. Chen Z.-Z. Zhou X.-F. Yin H.-B. Li X. Xin X.-F. (2009). Overexpression of *SOS* (Salt Overly Sensitive) Genes Increases Salt Tolerance in Transgenic Arabidopsis. Mol. Plant 2, 22–31. 10.1093/mp/ssn058 19529826 PMC2639737

[B73] YongChun G. PengJie W. Di C. YuCheng Z. XueJin C. NaiXing Y. (2019). Genome-wide Identification and Expression Analysis of *SRO* Gene Family in Camellia Sinensis. J. Tea Sci. 39, 392–402. 10.3969/j.issn.1000-369X.2019.04.004

[B74] You J. Zong W. Du H. Hu H. Xiong L. (2014). A Special Member of the rice SRO Family, *OsSRO1c*, Mediates Responses to Multiple Abiotic Stresses through Interaction with Various Transcription Factors. Plant Mol. Biol. 84, 693–705. 10.1007/s11103-013-0163-8 24337801

[B75] Zhang L. Zhou D. Hu H. Li W. Hu Y. Xie J. (2019). Genome-wide Characterization of a SRO Gene Family Involved in Response to Biotic and Abiotic Stresses in Banana (*Musa Spp*). BMC Plant Biol. 19, 211. 10.1186/s12870-019-1807-x 31113386 PMC6530135

[B76] Zhang J. (2003). Evolution by Gene Duplication: An Update. Trends Ecol. Evol. 18, 292–298. 10.1016/S0169-5347(03)00033-8

[B77] Zhao J. Zhou H. Li X. (2013). UBIQUITIN-SPECIFIC PROTEASE16 Interacts with a HEAVY METAL ASSOCIATED ISOPRENYLATED PLANT PROTEIN27 and Modulates Cadmium Tolerance. Plant Signaling Behav. 8, e25680. 10.4161/psb.25680 PMC4091083 23857362

