# Supplementary figures and images for: Genome-Wide Identification and Evolutionary Analysis of the SRO Gene Family in Tomato
Source: Front Genet. 2021 Sep 21;12:753638. doi: 10.3389/fgene.2021.753638 (PMC8490783; doi:10.3389/fgene.2021.753638)

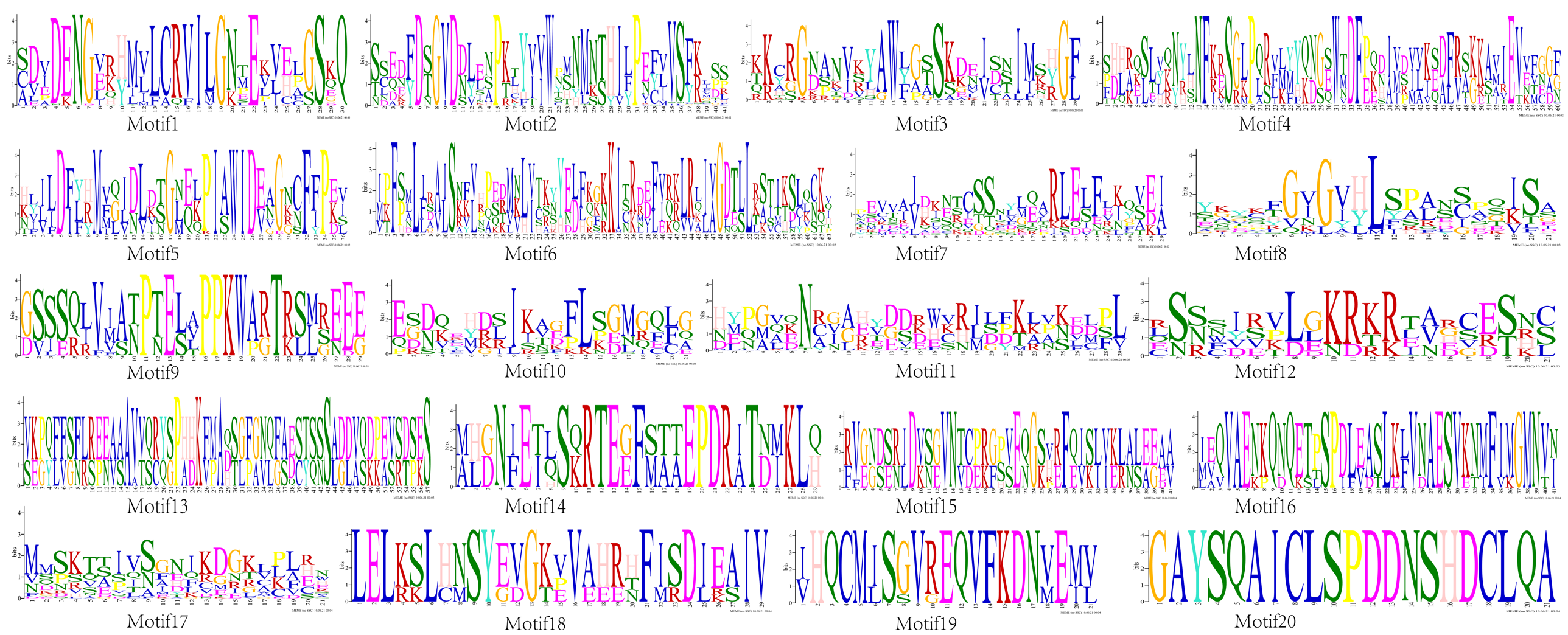

Supplement: Supplementary file 3 [file Image2.TIF]

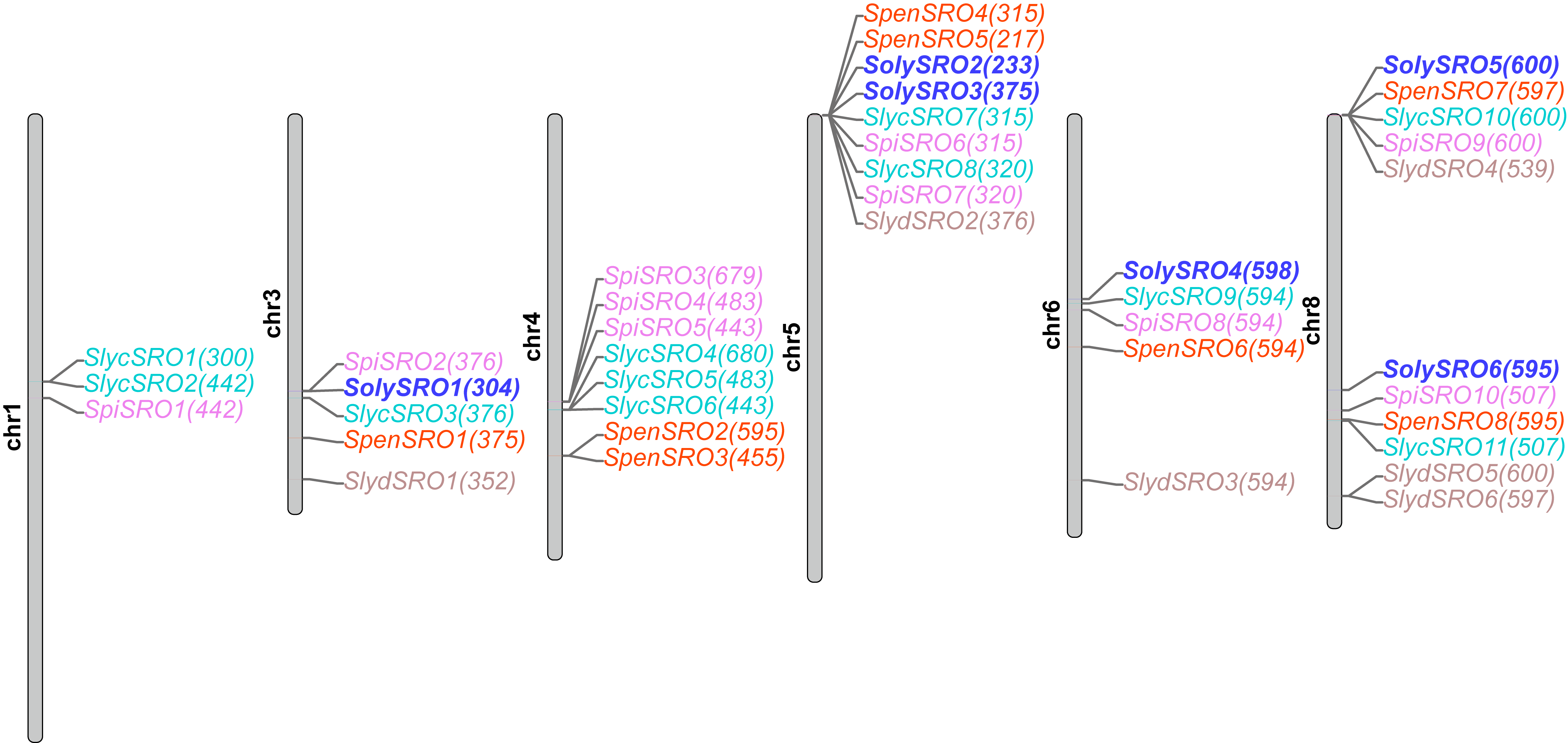

Supplement: Supplementary file 4 [file Image1.TIF]
